# Multi-omics analysis provides insights into the mechanism underlying fruit color formation in *Capsicum*

**DOI:** 10.3389/fpls.2024.1448060

**Published:** 2024-11-06

**Authors:** Zhao Song, Xiaowan Xu, Xiao Chen, Jingjing Chang, Jing Li, Jiaowen Cheng, Baige Zhang

**Affiliations:** ^1^ Guangdong Key Laboratory for New Technology Research of Vegetables, Vegetable Research Institute, Guangdong Academy of Agricultural Sciences, Guangzhou, China; ^2^ College of Horticulture, South China Agricultural University, Guangzhou, China

**Keywords:** *Capsicum*, fruit color, transcriptome, widely targeted metabolome, quantitative determination, whole-genome re-sequence

## Abstract

Fruit color is a crucial attribute of fruit quality in peppers (*Capsicum* spp.). However, few studies have focused on the mechanism of color formation in immature pepper fruits. In this study, the light-yellow color observed in immature CSJ009 fruits compared to CSJ010 could be attributed to decreased chlorophyll and carotenoid pigments. Through integrated analysis of the transcriptome and metabolome of CSJ009 and CSJ010, we identified 23,930 differentially expressed genes (DEGs) and 345 differentially accumulated metabolites (DAMs). Furthermore, integrated analysis revealed a strong correlation between the *HCT-like* gene and metabolite MWS0178 (chlorogenic acid). Paraffin section assay revealed that the epidermal cells of immature CSJ010 fruits exhibited a more compact arrangement with significantly greater length than those of CSJ009. Quantitative determination of carotenoids showed that lutein emerged as the predominant carotenoid in immature pepper fruits. Additionally, missense mutation of *LCYB2* is likely to lead to a decrease in *β*-carotene content in immature CSJ009 fruits, whereas CCS may directly catalyze the conversion of lycopene to *β*-carotene in mature fruits. The null mutation in *CCS* promoted the biosynthesis of *β,ϵ*-branch carotenoids leading to lutein being the most abundant carotenoid found in orange CSJ010 fruits. These findings provide important insights into the mechanism underlying color formation in pepper fruits and establish a foundation for the further exploration of color-related genes.

## 1 Introduction

Fruit color is a crucial trait for assessing fruit quality in peppers (*Capsicum* spp.). The colors of immature pepper fruit primarily include green, purple, and black, which can be attributed to the accumulation of various pigments such as chlorophylls, flavonoids, and carotenoids (Lightbourn et al., 2008). Chlorophylls are localized within chloroplasts, whereas flavonoids are found in cell vacuoles (Lightbourn et al., 2008). Carotenoids are contained within chromoplasts that develop from pre-existing chloroplasts during fruit ripening (Egea et al., 2011). The green color typically arises from the accumulation of chlorophylls, along with carotenoids, such as lutein, *β*-carotene, and violaxanthin (Deli et al., 1996; Paran and van der Knaap, 2007). Purple and black fruits mainly contain anthocyanins, chlorophyll, *β*-carotene, lutein, and violaxanthin (Lightbourn et al., 2008).

During the ripening process, the color of pepper fruit gradually transitions to red, orange, or yellow (Brand et al., 2012), which is determined by the amount and composition of carotenoids accumulated within chromoplasts (Ha et al., 2007). In red fruits, there is a significant increase in the total carotenoid content (Matus et al., 1991), and capsanthin is the predominant carotenoid (Bouvier et al., 1994), followed by *β*-cryptoxanthin, *β*-carotene, capsorubin, zeaxanthin, and antheraxanthin (Wahyuni et al., 2011); whereas chlorophyll levels decrease to zero (Deli et al., 1996). Yellow fruits do not contain detectable levels of capsanthin and capsorubin (Wahyuni et al., 2011), mainly due to the accumulation of *α*- and *β*-carotene, zeaxanthin, *β*-cryptoxanthin (Gómez-García and Ochoa-Alejo, 2013), violaxanthin, and lutein (Wahyuni et al., 2011). The orange-colored fruits primarily arise from the accumulation of *β*-carotene (Guzman et al., 2010), while also contain low but significant levels of red carotenoids, such as capsanthin, *β*-cryptoxanthin, and antheraxanthin (Wahyuni et al., 2011).

Chlorophyll is the most abundant tetrapyrrole molecule in photosynthetic organisms (Masuda and Fujita, 2008). Glutamyl-tRNA is activated by glutamyl-tRNA reductase (HEMA) to synthesize 5-aminolevulinic acid (ALA), the initial precursor of chlorophyll biosynthesis, which forms protoporphyrin IX (Masuda and Fujita, 2008). Mg-chelatase insert Mg^2+^ into protoporphyrin IX to produce Mg-protoporphyrin IX, which catalyzes the formation of chlorophyll *a* by a series of enzymes, such as magnesium chelatase subunit H (CHLH), magnesium protoporphyrin IX methyltransferase (CHLM), Mg-protoIX methyl ester oxidative cyclase (CRD), Genomes uncoupled 4 (GUN4), and protochlorophyllide oxidoreductase (POR). A portion of chlorophyll *a* undergoes conversion to chlorophyll *b* through the activity of chlorophyllide a oxygenase (CAO) (Masuda and Fujita, 2008). In *Capsicum*, *Capana10g001710* (*CaPP2C35*) has been identified as controlling the light-green immature fruit color (Wu et al., 2022). In addition, two quantitative trait loci (QTLs) *pc8.1* and *pc10.1* (Brand et al., 2012), along with three transcription factors (TFs), *Lsd one like1* zinc finger (*LOL1*) (Borovsky et al., 2019), *Golden2-like* (*GLK2*) (Brand et al., 2014) and *Arabidopsis pseudo-response regulator2* (*APRR2*) (Jeong et al., 2020; Lee et al., 2020; Pan et al., 2013) have been reported to regulate immature fruit color via modulating chlorophyll content and chloroplast development. In our recent study, we successfully mapped the locus *ly* responsible for the light-yellow immature fruit color into a 6.51Mbp region on chromosome 9 (Song et al., 2022).

Flavonoids are polyphenolic compounds widespread in the plant kingdom (Gil and Couto, 2013). To date, approximately 9000 different flavonoids have been found in all higher plants (Ferrer et al., 2008), which can be classified into six subclasses: flavonols, flavones, isoflavones, flavanones, anthocyanidins, and flavanols (Manach et al., 2004). Flavonoids are synthesized via from phenylalanine phenylpropanoid and flavonoid pathways. Phenylalanine is catalyzed by enzymes such as phenylalanine lyase (PAL), cinnamic acid 4-hydroxylase (C4H), and 4-coumadin-CoA ligase (4CL) to produce *p*-coumaroyl-CoA as the initial substrate for flavonoid synthesis (Liu et al., 2020). On one hand, *p*-coumaroyl-CoA undergoes enzymatic reactions involving chalcone synthase (CHS), chalcone isomerase (CHI), flavanone 3-hydroxylase (F3H), flavonoid-3’-hydroxylase (F3’H), and flavonoid 3’,5’-hydroxylase (F3’5’H) to generate dihydroflavonols (Liu et al., 2019). On the other hand, *p*-coumaroyl-CoA can also be converted to caffeoyl-CoA by shikimate *O*-hydroxycinnamoyltransferase (HCT) and coumaroylquinate 3-monooxygenase (C3H), which is subsequently activated by caffeoyl-CoA *O*-methyltransferase (CCOAOMT) to form feruloyl-CoA as an intermediate for dihydroflavonol biosynthesis (Pascual et al., 2016; Zhang et al., 2017). Dihydroflavonols are transformed into various metabolites via dihydroflavonol 4-reductase (DFR) and flavonol synthase (FLS) (Zhou et al., 2020). DFR catalyzes the stereospecific reduction of dihydroflavonols to leucoanthocyanidins (Huang et al., 2012; Martens et al., 2003). FLS converts dihydroflavonols into flavonols, which compete with DFR for common substrates at a key branch in the flavonoid pathway (Luo et al., 2016). Regulation of this complex flavonoid biosynthesis process relies mainly on MYB proteins (Nabavi et al., 2020). In *Capsicum*, anthocyanin accumulation in immature fruit is controlled by a MYB TF homologous to *Anthocyanin2* (*An2*) found in *Petunia* (Borovsky et al., 2004).

Carotenoids are polyisoprenoid compounds that can be classified as carotenes and xanthophylls (Rodríguez-Bernaldo de Quirós and Costa, 2006). To date, over 700 distinct carotenoids have been characterized (Nishino et al., 2009). Geranylgeranyl diphosphate (GGPP) is a common substrate used for the biosynthesis of carotenoids and chlorophylls (Wang et al., 2019). Phytoene synthase (PSY) catalyzes the initial committed condensation step from GGPP to generate colorless C40 phytoene, thereby directing metabolic flux towards carotenoid biosynthesis (Wang et al., 2019). Subsequently, phytoene is converted into acyclic lycopene by a series of key enzymes including phytoene desaturase (PDS), *ζ*-carotene isomerase (Z-ISO), *ζ*-carotene desaturase (ZDS), and carotenoid isomerase (CRTISO) (Wang et al., 2019). All-*trans*-lycopene undergoes cyclization by lycopene *β*-cyclases (LCYBs) to produce all-*trans*-*β*-carotene (*β,β*-branch), which is further catalyzed by enzymes such as *β*-carotene hydroxylase (CHYB), zeaxanthin epoxidase (ZEP), violaxanthin de-epoxidase (VDE), and capsanthin-capsorubin synthase (CCS), to form capsanthin and capsorubin (Yuan et al., 2015). All-*trans*-*β*-carotene can also be converted into 9-*cis*-*β*-carotene via *β*-carotene isomerase D27 (D27), thus directing metabolic flux towards strigolactones biosynthesis (Alder et al., 2012). On the other hand, the open end of lycopene can also be cyclized by lycopene *ϵ*-cyclase (LCYE) and LCYB to form *α*-carotene (*β,ϵ*-branch). This *α*-carotene then undergoes cyclization by *β*-hydroxylase (HYDB) and carotene *ϵ*-monooxygenase (CYP97C, namely LUT1) to produce lutein (Yuan et al., 2015). The degradation of carotenoids involves carotenoid cleavage dioxygenase (CCD) and 9-*cis* epoxy carotenoid cleavage dioxygenase (NCED) enzymes (Zhang et al., 2020). In pepper fruit, *β*-carotene and zeaxanthin are synthesized throughout the ripening process, whereas capsanthin in the *β,β*-branch is synthesized only in mature fruits, and lutein in the *β*,*ϵ*-branch is usually found in immature fruits (Wang et al., 2019).

However, to date, few studies have focused on identifying the transcriptional regulators and metabolites that influence immature fruit color in pepper. In this study, we conducted an integrative analysis of the transcriptome and metabolome to elucidate the mechanism underlying fruit color formation in *Capsicum*. Our integrative analysis revealed a strong correlation between the *HCT-like* gene and metabolite MWS0178. The qRT-PCR assay demonstrated negligible expression of *HCT-like* in immature light-yellow CSJ009 fruits, likely due to a null mutation identified through whole-genome re-sequencing (WGRS) analysis. Furthermore, WGRS analysis identified several mutations in carotenoid-biosynthesis genes, including *LCYB2*, *APRR2* and *CCS*. Quantitative determination provided robust evidence to validate the function of these color-related genes and clearly illustrated the differences in carotenoid concentrations between CSJ009 and CSJ010 fruits at different developmental stages. Our findings not only provide novel insights into the mechanism underlying color formation in pepper fruits but also lay a foundation for further exploration of color-related genes.

## 2 Materials and methods

### 2.1 Plant growth and sampling

**Figure 1:**
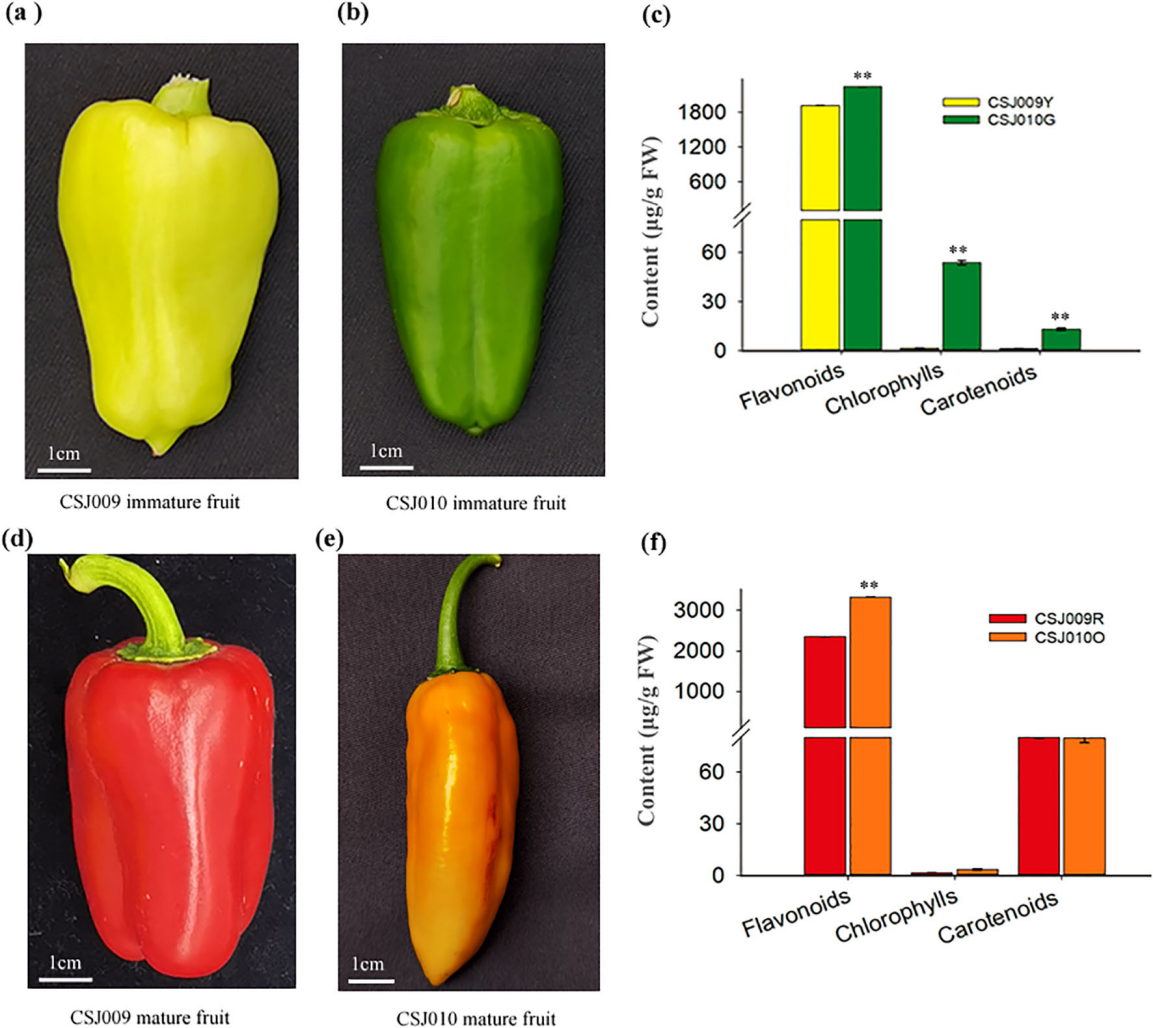
Fruit color and pigment content in CSJ009 and CSJ010. Subfigures **(A)** and **(B)** present the immature fruit colors of CSJ009 and CSJ010, respectively. Subfigures **(D)** and **(E)** show the mature fruit colors of CSJ009 and CSJ010, respectively. Subfigures **(C)** and **(F)** present the variation of chlorophylls, flavonoids, and carotenoids in immature and mature fruits, respectively. The data are presented as mean ± standard deviation (n = 12). Different capital letters indicate significant difference (**P < 0.01) in pigment content between the two samples in Student’s *t*-test.

For this study, two independent highly inbred lines of *C. annuum*, namely ‘CSJ009’ and ‘CSJ010’ were used after undergoing multiple generations of self-crossing. The immature fruits (40 d post-anthesis) of CSJ009 and CSJ010 are light-yellow (**Figure 1A**; hereafter referred to as CSJ009Y) and green (**Figure 1B**; hereafter referred to as CSJ010G), respectively. Upon maturation (80 d post-anthesis), the fruit color changed to red for CSJ009 (**Figure 1C**; hereafter referred to as CSJ009R) and orange for CSJ010 (**Figure 1D**; hereafter referred to as CSJ010O). Both inbred lines were cultivated under identical environmental conditions with consistent management practices in an open field located at the DaFeng Research Base, Vegetable Research Institute, Guangdong Academy of Agricultural Sciences, Guangzhou, China.

The young leaves of the two inbred lines were collected for WGRS. As a biological replicate of immature-stage samples, fruit peels (1 cm wide in the middle) from three fruits of different individual plants per inbred line were collected 40 d post-anthesis on November 4, 2020. The mature-stage samples were prepared 80 d post-anthesis using the aforementioned method on December 14, 2020. Three biological replicates per inbred line were sampled in both the immature and mature stages, resulting in a total of 12 samples that were submitted to biotech companies for transcriptome, metabolome, and pigment analyses.

### 2.2 Determination of chlorophylls, carotenoids and flavonoids

The chlorophyll and carotenoid contents were quantified following the methodology described by Wang et al. (2020) and Song et al. (2022). The total flavonoid content was determined using a NaNO_2_-Al(NO_3_)_3_-NaOH colorimetric assay (Jang et al., 2024).

### 2.3 Scanning and transmission electron microscopy

The pericarps of fresh fruit from each inbred line were dissected into small fragments and fixed using a stationary liquid 40 d post-anthesis. Samples were cut into paraffin sections, stained with toluidine blue, and observed using a PANNORAMIC microscope (3DHISTECH, Hungary). Another set of samples was visualized using a HITACHI HF7700 system (Hitachi, Japan) for the TEM assay.

### 2.4 Metabolome analysis

Metabolite profiling was conducted by the Wuhan Metware Biotechnology Co. Ltd. (Wuhan, China). Carotenoid extracts were quantified using a UPLC-APCI-MS/MS system (ExionLC™ AD; MS, Applied Biosystems 6500 Triple Quadrupole). The sample was freeze-dried, ground into a powder (30 Hz, 1.5 min), and stored at -80°C. The powder (50 mg) was weighed and extracted with 0.5 mL mixed solution of *n*-hexane: acetone: ethanol (1:1:1, v/v/v). The extract was vortexed for 20 min at room temperature. The supernatants were collected after centrifuged at 12000 r/min for 5 min at 4°C. The residue was reextracted by repeating the aforementioned steps under the same conditions. The mixture was evaporated to dryness, and reconstituted in a mixed solution of MeOH/MTBE (1:1, v/v). The solution was filtered through a 0.22 μm membrane filter for further LC-MS/MS analysis (Inbaraj et al., 2008).

Widely targeted metabolomic analysis of metabolites (mainly flavonoids) was carried out using a UPLC-ESI-MS/MS system (UPLC, Shim-pack UFLC SHIMADZU CBM30A system; MS, Applied Biosystems 4500 QTRAP). In brief, the freeze-dried fruit peel was crushed into powder using a mixer mill (MM 400, Retsch) with zirconia bead for 1.5 min at 30 Hz. Subsequently, 100 mg of the powder was extracted overnight at 4°C with 0.6 mL 70% aqueous methanol. After centrifugation at 10,000 × *g* for 10 min, the extracts were absorbed and filtered before LC-MS/MS analysis (Chen et al., 2013). Metabolite quantification was performed in the multiple reaction monitoring (MRM) mode. A combination of principal component analysis (PCA), fold change (FC) and orthogonal projections to latent structures discriminant (OPLSDA) analyses was used to identify differentially accumulated metabolites (DAMs) based on three criteria: VIP (variable importance in projection) ≥ 1, FC ≥ 2 or ≤ 0.5, and *P* value ≤ 0.05 (Yun et al., 2015).

### 2.5 Transcriptome analysis and WGRS analysis

Transcriptome profiling was performed using Biomarker Technologies Co. Ltd. (Beijing, China). Total RNA was extracted from 12 frozen peel samples using TRIZOL reagent (Invitrogen). Approximately 1 μg of RNA per sample was used as the input material for RNA sample preparations. Sequencing libraries were generated and index codes were added to attribute sequences to each sample. Purified mRNAs were cleaved into small fragments using divalent cations under elevated temperatures and reverse-transcribed into first-strand cDNAs using random hexamer primers and M-MuLV Reverse Transcriptase. Subsequently, double-stranded cDNAs were synthesized, purified, end-repaired, and ligated to the adaptors. Finally, cDNA libraries were constructed by PCR for paired-end sequencing. After clustering the index-coded samples, library preparations were sequenced on an Illumina XtenPE150 platform.

Hisat2 (Daehwan et al., 2015) was used to map the clean reads onto *C. annuum* Zunla-1 genome sequence (Qin et al., 2014). Picard and samtools (Li et al., 2009) were used to sort and remove duplicated reads and merge the alignment results of each sample. Gene function was annotated using BLAST (Altschul et al., 1997) by aligning their sequences to GO, COG, Pfam, KEGG, NR, and Swiss-Prot databases. The gene expression level was calculated and normalized using the FPKM (Fragments Per Kilobase of exon model per Million mapped fragments) method (Li and Dewey, 2011). To control the false discovery rate (FDR), Benjamini and Hochberg’s approach (Benjamini and Hochberg, 1995) was applied to adjust the resulting *P* values, while DESeq2 (Love et al., 2014) was utilized to identify DEGs based on two criteria: FC ≥ 2 or ≤ 0.5 and FDR (false discovery rate) ≤ 0.01). The identified DEGs were further subjected to the GO, COG, and KEGG databases for enrichment analysis. To explore the correlation between these DEGs and DAMs, canonical correlation analysis (CCA) and a network graph of correlation were performed.

Total genomic DNA was extracted from the young leaves of the two inbred lines using an improved CTAB method (Allen et al., 2006). DNA was randomly fragmented and fragments ranging from 300 to 400 bp were selected for amplification. These amplified fragments were used to construct paired-end libraries, which were subjected to paired-end sequencing on an Illumina HiSeq ™ 2500 platform. Finally, the obtained DNA sequences were optimized, aligned, and annotated using the same procedure as that used for the RNA sequencing.

### 2.6 Quantitative real-time PCR validation

Total RNA was extracted from the fruit peels of CSJ009 and CSJ010 at 40 d post-anthesis using TRIzol reagent (Invitrogen) and then purified with a DNA-free RNA kit. First-strand cDNA was synthesized using the Fermentas RevertAid First Strand cDNA Synthesis Kit. *Ca-Actin* (*GQ339766*) served as a reference gene for the normalization of gene expression (Wan et al., 2011). All primers were designed using Primer Premier 6.0 (PREMIER Biosoft, USA), and their details are provided in **Supplementary Table S1**. qRT-PCR was conducted using a Roche Light Cycler 2.10 with a 2×SYBR Green I PCR Master Mix according to the following procedure: 95°C for 3 min; 39 cycles of 95°C for 10 s, 58°C for 30 s; and 72°C for 4 s, and quantitative data of gene expression were analyzed using the 2^-ΔΔ^ *^C^* ^T^ method (Livak and Schmittgen, 2001).

### 2.7 Statistical analysis

All experiments were performed in triplicate, and the results are expressed as mean ± SE. Analysis of variance was performed using SPSS (version 22.0; SPSS Institute Inc., United States), and treatment means were compared using Student’s *t*-test. Two subfigures (C and F) in **Figure 1** were prepared using SigmaPlot 14.0 (Systat Software, Inc., Germany). Other statistical charts were generated using Microsoft Excel 2013 (Microsoft Corporation, United States).

## 3 Results

### 3.1 Variation of pigment content

CSJ010 bears green immature and orange mature fruits, whereas CSJ009 bears light-yellow immature and red mature fruits (**Figure 1**). Chlorophyll was barely detected in CSJ009R and CSJ010O. In contrast, the levels of chlorophylls in CSJ010G (53.87 μg g^-1^ FW) were 34 times higher compared to those in CSJ009Y (1.61μg g^-1^ FW). Similarly, the total carotenoid content of CSJ010G (13.21 μg g^-1^ FW) was significantly higher (*P* < 0.01) than that of CSJ009Y (1.43 μg g^-1^ FW). However, the total carotenoid level in CSJ009R (80.05 μg g^-1^ FW) was comparable to that in CSJ010O (79.74 μg g^-1^ FW). The total flavonoid content of CSJ010G (2242.79 μg g^-1^ FW) was 17% higher than that of CSJ009Y (1919.37 μg g^-1^ FW).

### 3.2 Phenotype analysis of immature fruit skin in pepper

**Figure 2:**
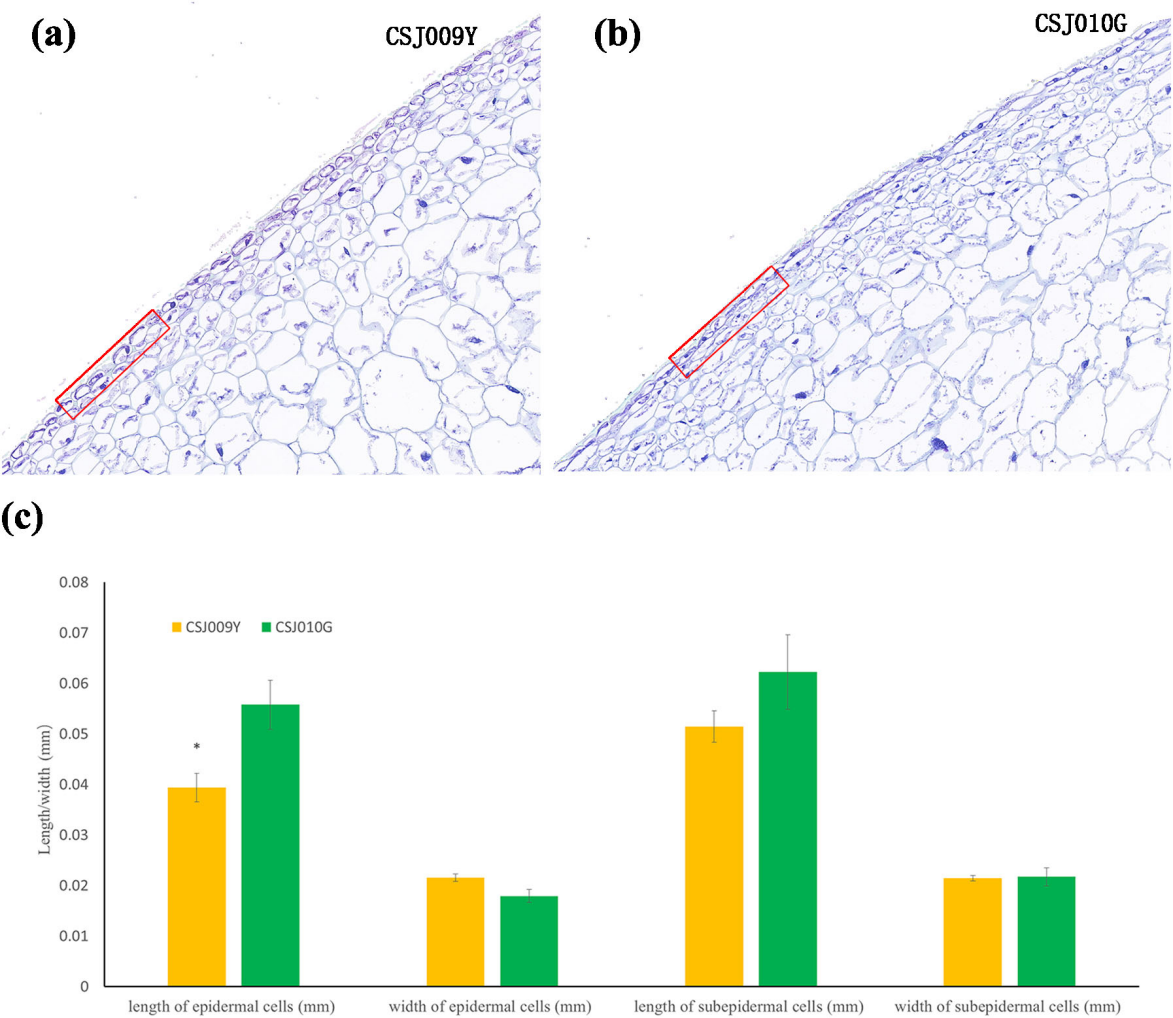
Paraffin section assay of the fruit skin in CSJ009Y and CSJ010G. Subfigures **(A, B)**, the arrangement of skin cells. Subfigure **(C)** shows the difference in the length and width of the skin cells between CSJ009Y and CSJ010G. *P < 0.05 in Student’s *t*-test.

Paraffin section assay revealed that the epidermal cells of CSJ010G exhibit a more compact arrangement (**Figures 2A, B**) and significantly greater length (**Figure 2C**) than those of CSJ009Y. The difference between CSJ009Y and CSJ010G in the length and width of subepidermal cells was smaller than that of epidermal cells (**Figure 2C**).

**Figure 3:**
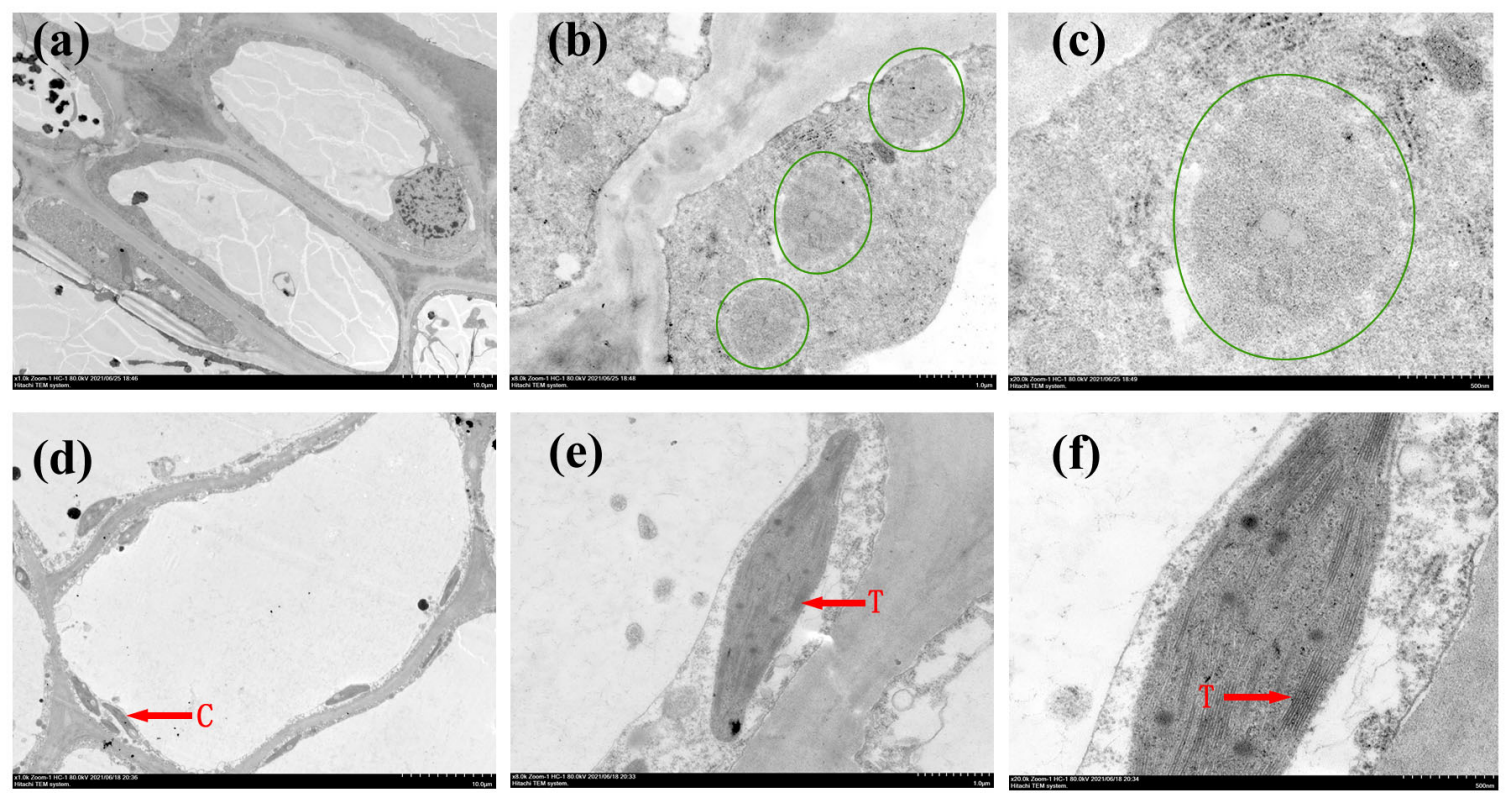
Transmission electron microscopy observations of the fruit skin in CSJ009Y and CSJ010G. Subfigures **(A–C)**, TEM images of CSJ009 pericarp cells. Subfigures **(D–F)**, TEM images of CSJ010 pericarp cells. The tissue in the green circle represents a proplastid. ‘C’ represents chloroplast. ‘T’ represents thylakoid. Scale bar in **(A)** and **(D)** 10.0 μm, **(B)** and **(E)** 1.0 μm, **(C)** and **(F)** 500.0 nm.

We also examined the inner structures of plastids in immature fruits using TEM. Chloroplasts exhibiting the characteristic thylakoid membrane structure were clearly observed in CJS010G (**Figures 3D–F**), whereas they were barely detectable even in the 20× magnified images in CSJ009Y (**Figures 3A–C**). The presence of a greater number of chloroplasts and thylakoids within the pericarp cells of CSJ010G, compared to CSJ009Y, suggests a higher accumulation of chlorophyll in the immature fruit skins of CSJ010 than those of CSJ009, which is consistent with the pigment substance determination results.

### 3.3 Identification of differently expressed genes

After conducting mRNA sequencing on 12 samples, a total of 91.79 Gbp clean data were generated, with a minimum of 6.77 Gbp per sample. A total of 8,152 new genes were discovered, of which 79.8% were successfully annotated. Through differential expression analysis, we identified 23,930 DEGs across the four groups (**Supplementary Table S2**), with DEG numbers ranging from 1,212 to 5,744 between any two groups. From these DEGs, we classified 726 TFs into 59 families, among which AP2/ERF-ERF, bHLH, C2H2, MYB, and B3 were the top five TF families (**Supplementary Figure S1**).

In the immature-fruit group (CSJ009Y vs CSJ010G), we found a total of 2,520 DEGs, comprising 1,332 upregulated genes and 1,188 downregulated genes. The volcano plot clearly illustrates the differences in the gene expression levels between the two groups (**Supplementary Figure S2**). KEGG analysis revealed that 441 DEGs were enriched in 108 pathways, with 23 DEGs specifically identified within the three color-related pathways (**Supplementary Table S3**). Additionally, in the mature-fruit group (CSJ009R vs. CSJ010O), we identified a set of 2,280 DEGs, comprising 1,212 upregulated and 1,068 downregulated genes.

**Figure 4:**
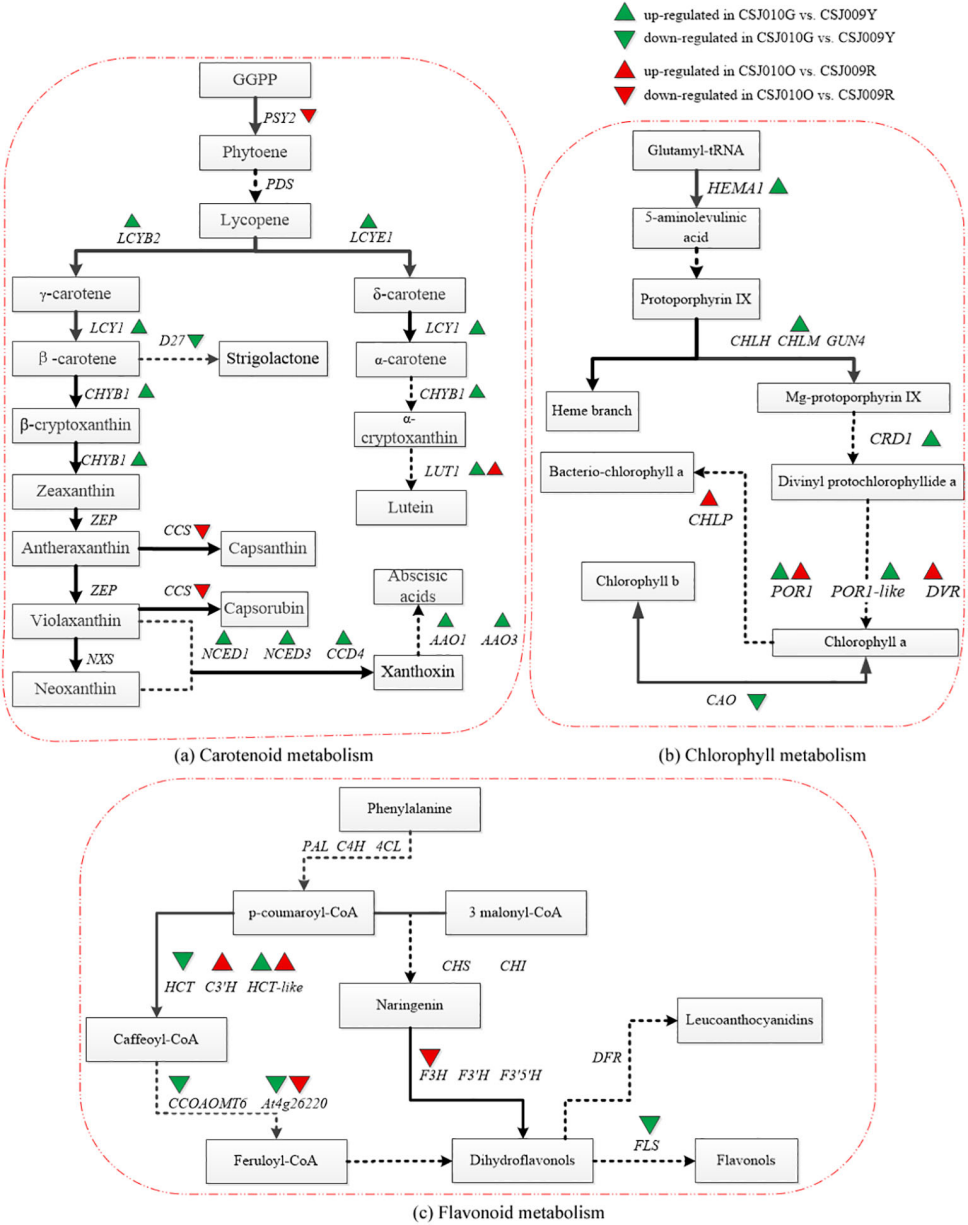
Pathway map of DEGs related to chlorophyll, carotenoid and flavonoid metabolism in KEGG annotation analysis. Subfigure **(A)**, the pathway map of carotenoid metabolism was plotted according to the differential expression analysis and KEGG annotation analysis with reference to Yuan’s study (Yuan et al., 2015). *PSY2* (*Phytoene desaturase 2*, *Capana02g002284*), *PDS* (*Phytoene desaturase*), *LCYB2* (*Lycopene β-cyclase*, *Capana10g002320*), *LCYE1* (*LCYE: Lycopene ϵ-cyclase*, *Capana09g000177*), *LUT1* (*Carotene epsilon-monooxygenase*, *Capana10g001912*), *ZEP* (*Zeaxanthin epoxidase*), *CCS* (*Capsanthin-capsorubin synthase*, *Capana06g000615*), *NXS* (*Neoxanthin synthase*), *CCD4* (*Carotenoid cleavage dioxygenase 4*, *Capana01g000948*), *NCED1* (*9-cis-epoxy carotenoid dioxygenase 1*, *Capana00g003114*), *NCED3* (*9-cis-epoxy carotenoid dioxygenase 3*, *Capana01g003704*), *AAO1* (*Indole-3-acetaldehyde oxidase 1*, *Capana11g000423*), *AAO3* (*Abscisic-aldehyde oxidase 3*, *Capana11g000422*), *CHYB1* (*β-carotene hydroxylase1*, *Capana03g002170*). Subfigure **(B)**, the pathway map of chlorophyll metabolism was plotted according to the differential expression analysis and KEGG annotation analysis with reference to Masuda’s study (Masuda and Fujita, 2008). *CAO* (*Chlorophyllide a oxygenase*, *Capana06g001725*), *CHLM* (*Magnesium protoporphyrin IX methyltransferase*, *Capana03g000633*), *CRD1* (*Magnesium-protoporphyrin IX monomethyl ester*, *Capana10g002504*), *POR1* (*Protochlorophyllide oxidoreductase1*, *Capana00g004560*), *POR1-like* (*Protochlorophyllide oxidoreductase 1-like*, *Capana10g000065*), *HEMA1* (*Glutamyl-tRNA reductase1*, *Capana04g000804*), *CHLP* (*Geranylgeranyl diphosphate reductase*, *Capana03g000791*), *DVR* (*Divinyl chlorophyllide a 8-vinyl-reductase*, *Capana01g003169*), *CHLH* (*Magnesium chelatase subunit H*), *GUN4* (*Genomes uncoupled4*). Subfigure **(C)**, the pathway map of flavonoid metabolism was plotted according to the differential expression analysis and KEGG annotation analysis with reference to Yuan’s study (Yuan et al., 2015). *HCT* (*Shikimate O-hydroxycinnamoyltransferase*, *Capana03g000549*), *HCT-like* (*Shikimate O-hydroxycinnamoyltransferase -like*, *Capana09g000120*), *PAL* (*Phenylalanine lyase*), *C4H* (*Cinnamic acid 4-hydroxylase*), *4CL* (*4-coumadin-CoA ligase*), *CHS* (*Chalcone synthase*), *CHI* (*Chalcone isomerase*), *F3H* (*Flavanone 3-hydroxylase*, *Capana02g002586*), *F3’H* (*Flavonoid-3’-hydroxylase*), *F3’5’H* (*Flavonoid 3’,5’-hydroxylase*), *C3’H* (*Coumaroylquinate 3’-monooxygenase*, *Capana10g002482*), *CCOAOMT* (*Caffeoyl-CoA O-methyltransferase*, *Capana08g002351*), *At4g26220* (*Caffeoyl-CoA O-methyltransferase At4g26220 isoform X1*, *Capana00g004448*), *DFR* (*Dihydroflavonol 4-reductase*), *FLS* (*Flavonol synthase*, *Capana09g002174*). The green upward or downward triangle indicates that the gene was upregulated or downregulated in CSJ010G compared to CSJ009Y. The red upward or downward triangle indicates that the gene was upregulated or downregulated in CSJ010O compared to CSJ009R.

As shown in **Figure 4**, the majority of genes associated with chlorophyll and carotenoid synthesis (e.g., *HEMA1*, *CHLM*, *CRD1*, *POR1*, *LCYB2*, *LCYE1*, *CHYB1* and *LUT1*) were upregulated in CSJ010G compared to CSJ009Y. Interestingly, most genes involved in flavonoid-biosynthesis (e.g., *HCT*, *FLS*, *CCOAOMT*, and *At4g26220*) were downregulated in CSJ010G, except for *HCT-like*. Furthermore, both *PSY2* and *CCS* involved in carotenoid synthesis were downregulated in mature orange CSJ010O fruit relative to red CSJ009R fruit. Three genes (*CHLP*, *POR1 and DVR*) implicated in chlorophyll synthesis were upregulated.

Color-related genes in immature fruits were the focus of our study. *PP2C35* was not detected in any of the four groups (**Table 1**). The expression levels of the three TFs were drastically downregulated (*P* < 0.01) in mature fruits compared to immature fruits, indicating their predominant expression in immature fruits. There were no significant differences in the expression levels of *GLK2* and *LOL1* between immature- and mature-fruit groups. *APRR2* expression was significantly higher (*P* < 0.01) in CSJ009 than in CSJ010.

**Table 1:** Differential expressions of *LOL1*, *GLK2*, *APRR2*, and *PP2C35*.

Gene name	CSJ009Y vs. CSJ009R	CSJ009Y vs. CSJ010G	CSJ010G vs. CSJ010O	CSJ009R vs. CSJ010O
*LOL1* (*Capana06g002446*)	down (-12.022)	normal (-0.912)	down (-7.235)	–
*GLK2* (*Capana10g000333*)	down (-8.065)	normal (-0.345)	down (-8.777)	–
*APRR2* (*Capana01g000809*)	down (-4.308)	down (-1.495)	down (-6.279)	down (-2.501)
*PP2C35* (*Capana10g001710*)	–	–	–	–

The term ‘down’ indicates that the gene was downregulated in the latter sample compared with the former. The term ‘normal’ indicates no differential expression. The symbol ‘-’ depicts that no expression was detected. The values in parentheses denote the logarithm of fold change (log2FC).

### 3.4 Identification of differently accumulated metabolites

A total of 568 metabolites were identified using widely targeted metabolomic analysis, which can be classified into 11 known classes of metabolites (**Supplementary Figure S4**). The top five metabolites were flavonoids (18.8%), lipids (16.5%), amino acids (12.7%), phenolic acids (12.1%), and alkaloids (9.7%). Through a conjoint analysis of PCA, FC and OPLSDA, 345 metabolites were identified as DAMs among the four groups (**Supplementary Table S4**). Among the 345 DAMs, 86 and 121 were detected in the mature- and immature-fruit groups, respectively.

Eighteen differentially accumulated flavonoids (DAFs) associated with pepper fruit color were identified as representative DAMs (**Supplementary Table S5**). In the immature-fruit group, most flavones and flavonols were upregulated in CSJ009Y compared with CSJ010G, except for epicatechin glucoside, kaempferol-3-*O*-(6’’-malonyl) galactoside, and kaempferol-3-*O*-galactoside (trifolin). In the mature-fruit group, half of the DAFs were upregulated in CSJ009R vs. CSJ010O.

As depicted in the volcano plot (**Supplementary Figure S3A**), chlorogenic acid was the only metabolite downregulated in CSJ009Y vs. CSJ010G. Within the mature-fruit group, all top five DAMs were upregulated in CSJ009R vs. CSJ010O (**Supplementary Figure S3B**). Among the three color-related metabolic pathways, only DAMs and DEGs involved in the flavonoid biosynthesis pathway fulfilled the criteria for correlation analysis. As illustrated in **Supplementary Figure S3**, *Capana09g000120* was the exclusive gene that positively correlated with MWS0178 (chlorogenic acid).

### 3.5 Several mutations of color-related genes were identified via WGRS

**Figure 5:**
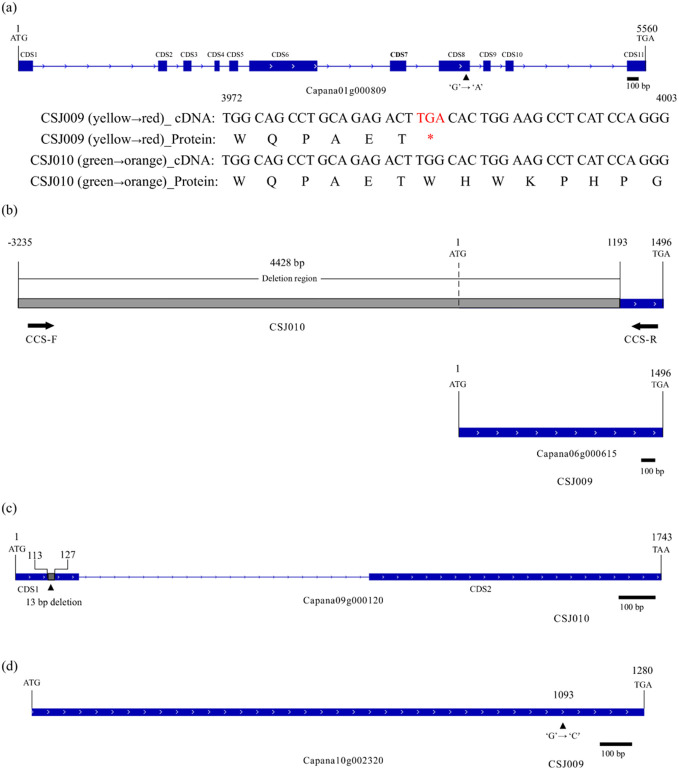
Mutations in *APRR2*, *CCS*, *HCT-like* and *LCYB2*. Subfigure **(A)**, Nonsense mutation of *APRR2* (*Capana01g000809*) in CSJ009. The symbol '*' means 'TGA' is a terminator. Subfigure **(B)**, Null mutation of *CCS* (*Capana06g000615*) in CSJ010. Subfigure **(C)**, Null mutation of *HCT-like* (*Capana09g000120*) in CSJ009. Subfigure **(D)**, Missense mutation of *LCYB2* (*Capana10g002320*) in CSJ009. Blue and gray rectangles indicate the coding and deletion region, respectively.

The differential expression of *GLK2*, *LOL1*, and *APRR2* in the transcriptome analysis promoted us to investigate their coding sequences. WGRS data revealed a G-to-A substitution in the coding DNA sequence (CDS) 8 region of *APRR2*, resulting in a premature stop codon in CSJ009 (**Figure 5A**), whereas no non-synonymous single nucleotide variants (SNV) were found in *PP2C35*, *GLK2*, and *LOL1*. The nonsense mutation observed in *APRR2* was identical to that reported previously in *C. annuum* IT158782 (Lee et al., 2020) and white-fruited pepper lines (Pan et al., 2013).

Several mutations in carotenoid-biosynthesis genes have been subsequently identified in re-sequencing data. A 4,428 bp deletion encompassing the promoter and coding regions of *CCS* (*Capana06g000615*) was detected in CSJ010 (**Figure 5B**). In the CDS1 region of *HCT-like* (*Capana09g000120*), a 13 bp coding sequence was deleted (**Figure 5C**). In addition, one non-synonymous SNV was observed in the coding regions of *LCYB2* (*Capana10g002320*, **Figure 5D**) and *PIF1* (*Capana09g001161*) in CSJ009. To verify these mutations, PCR amplification of related genes was performed for CSJ009 and CSJ010, and sequencing results showed that the amplified fragment sequences were consistent with the WGRS results, except for the *CCS* gene. We designed several primers for *CCS* gene but failed to amplify them successfully in CSJ010.

### 3.6 Identification of differently accumulated carotenoids

A total of 68 carotenoids were identified in the four samples (**Supplementary Table S6**). In immature fruits, 20 and 17 carotenoids were assayed in the CSJ009 and CSJ010, respectively. Lutein has emerged as the predominant carotenoid, constituting over 73% of total carotenoid content. Notably, lutein, neoxanthin, zeaxanthin, *β*-carotene, violaxanthin, and antheraxanthin ranked among the top six carotenoids in both accessions. The immature fruits of CSJ009 contained a significantly lower amount of total carotenoids (37.9 μg g^-1^ FW) than those of CSJ010 (360.48 μg g^-1^ FW), representing only 10.5% of its counterpart.

In mature fruits, 44 carotenoids were measured in both the inbred lines. However, the predominant carotenoid components exhibited significant differences (**Table 2**). The red CSJ009 fruits displayed the highest concentration of capsanthin (21.69%), followed by phytoene (20.99%), *β*-carotene (11.09%), zeaxanthin palmitate (4.75%), violaxanthin myristate (4.43%), and zeaxanthin dimyristate (4.37%). Lutein (39.9%) and phytoene (17.3%) were the major constituents of orange CSJ010 fruit, whereas violaxanthin myristate (11.82%), lutein palmitate (6.25%), lutein dimyristate (5.05%), and *α*-carotene (3.04%) followed. Capsanthin (only 0.087 μg g^-1^ FW) was barely detected in CSJ010O mature fruits, while the total carotenoid content of CSJ010O reached 613.67 μg g^-1^ FW, surpassing that of CSJ009R (481.46 μg g^-1^ FW). During the fruit maturation stages, there was an increase of 1170.47% and 70.24% in the total carotenoid content for CSJ009 and CSJ010 fruits, respectively.

**Table 2:** Quantitative determination of carotenoid content in CSJ009 and CSJ010 fruits.

Name	Ripening stage	Main carotenoids	Total carotenoids
CSJ009	Immature (Light-yellow)	Lutein (73.8%) Neoxanthin (5.59%) Zeaxanthin (5.34%) *β*-carotene (4.43%) Violaxanthin (3.42%) Antheraxanthin (1.9%)	37.9 μg g^-1^ FW
	Mature (Red)	Capsanthin (21.69%) Phytoene (20.99%) *β*-carotene (11.09%) Zeaxanthin palmitate (4.75%) Violaxanthin myristate (4.43%) Zeaxanthin dimyristate (4.37%) 5,6 epoxy-luttein dilaurate (4.08%) Zeaxanthin (4.06%)	481.46 μg g^-1^ FW
CSJ010	Immature (Green)	Lutein (76.97%) Neoxanthin (7.95%) *β*-carotene (7.59%) Violaxanthin (4.06%) Antheraxanthin (1.76%) Zeaxanthin (0.93%)	360.48 μg g^-1^ FW
	Mature (Orange)	Lutein (39.9%) Phytoene (17.3%) Violaxanthin myristate (11.82%) Lutein palmitate (6.25%) Lutein dimyristate (5.05%) *α*-carotene (3.04%) Violaxanthin (2.79%) Lutein dipalmitate (2.57%)	613.67 μg g^-1^ FW

As the fruit ripens, the amount and composition of carotenoids undergo drastic changes. A total of 28 differentially accumulated carotenoids (DACs) were detected in the four groups (**Supplementary Table S7**). Zeaxanthin dimyristate, violaxanthin myristate, and *β*-carotene exhibited remarkable increases of 617.83-fold, 72.93-fold and 31.83-fold, respectively, in CSJ009R vs. CSJ009Y. Conversely, lutein level decreased dramatically by 97.68% in CSJ009R compared to CSJ009Y; however, no significant change (FC = 0.88) was observed in CSJ010 fruit during this period. The levels of lutein dilaurate, lutein dimyristate, and violaxanthin myristate in CSJ010O were significantly increased by 677.41-fold, 528.23-fold, and 459.95-fold, respectively, compared to those in CSJ010G. In contrast to the upregulated expression of *β*-carotene in CSJ009R vs. CSJ009Y (FC = 31.8), the level of *β*-carotene in CSJ010O was only 41% of that in CSJ010G (FC = 0.41).

The composition and content of carotenoids in the fruits at the same growth-time fruits exhibited significant variations. In mature fruits, 27 DACs have been identified in CSJ009R and CSJ010O. Lutein, violaxanthin, and their fatty-acid esters (dilaurate, dimyristate, dipalmitate, myristate, myristate, and laurate) were upregulated. In contrast, zeaxanthin and its esters (palmitate, laurate, myristate, and dimyristate) were downregulated in this group. In particular, lutein increased by a remarkable 377-fold, whereas capsanthin decreased by an astonishing 1199-fold in CSJ010O compared to CSJ009R. In immature fruits, only two lutein esters (dilaurate and dimyristate) displayed down-regulation in CSJ010G vs. CSJ009Y.

### 3.7 qRT−PCR analysis of color-related genes

**Figure 6:**
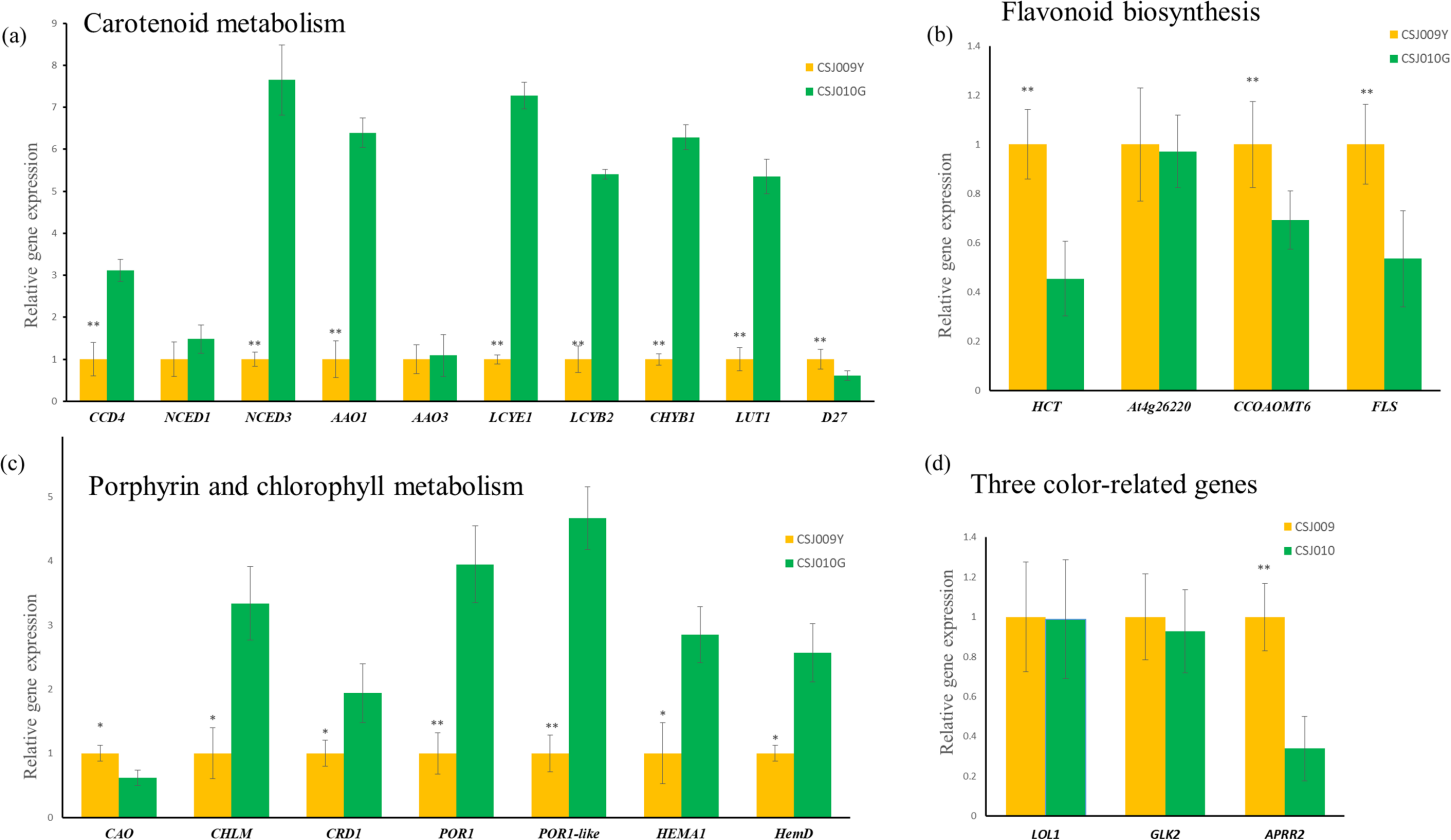
qRT-PCR expression analysis of color-related genes between CSJ009Y and CSJ010G. The X-axis genes depicted in subfigures **(A–C)** are interpreted as illustrated in **Figure 4**. Subfigure **(D)**, The X-axis represents genes: *Lsd one like1* (*LOL1*, *Capana06g002446*), *Golden2-like* (*GLK2*, *Capana10g000333*), *Arabidopsis pseudo response regulator2* (*APRR2*, *Capana01g000809*). The Y-axis in subfigures **(A–D)** represents the relative gene expression. Error bar is standard error. *P < 0.05 and **P < 0.01 in Student’s *t*-test.

qRT-PCR analyses of representative genes, including 22 DEGs enriched in three color-related metabolic pathways (**Supplementary Table S3**) and four color-related genes (**Table 1**), were performed for CSJ009Y and CSJ010G. The majority of chlorophyll and carotenoid biosynthesis genes exhibited upregulated expression levels in the immature fruits of CSJ010G compared to CSJ009Y (**Figure 6**), except for *CAO* and *D27*. Additionally, the expression of three carotenoid degradation genes (*CCD4*, *NCED1*, and *NCED3*) was also upregulated in CSJ010G vs. CSJ009Y. However, *APRR2* was significantly upregulated (*P* < 0.01) in CSJ009Y vs. CSJ010G, whereas almost no *PP2C35* expression was detected in CSJ009Y and CSJ010G. To confirm this observation, three independent qRT−PCR assays were repeated, which confirmed that *HCT-like* were highly expressed in CSJ010G but not in CSJ009Y. These results indicate that the downregulated expressions of genes involved in chlorophyll and carotenoid biosynthesis led to reduced levels of chlorophyll and carotenoids in CSJ009Y compared to CSJ010G, which is in agreement with the transcriptome data.

## 4 Discussion

Pepper (*Capsicum* spp.) is an appealing crop for fruit color research owing to its rich color. Fruit of the two inbred lines CSJ009 and CSJ010, exhibited distinct colors at both immature and ripe stages. According to the prevailing theory, the colors of immature and ripe pepper fruits are controlled by a different series of genes. Mature fruit color is governed by three loci: *y*, *c1*, and *c2* (Kormos and Kormos, 1960; Wang and Bosland, 2006). *CCS*, *APRR2*, and *PSY* have been identified as candidate genes for *y*, *c1*, and *c2*, respectively (Huh et al., 2001; Lee et al., 2020; Lefebvre et al., 1998). In contrast, green to ivory immature fruit colors are attributed to three loci: *sw1*, *sw2*, and *sw3* (Lightbourn et al., 2008). However, no candidate genes were identified for these three loci. Only two QTLs *pc8.1* (renamed *pc1*), *pc10.1* (Borovsky et al., 2019; Brand et al., 2012), along with four genes, including *GLK2* (Brand et al., 2014), *LOL1* (Borovsky et al., 2019), *APRR2* (Jeong et al., 2020; Lee et al., 2020), and *PP2C35* (Wu et al., 2022) are thought to regulate immature fruit color. In this study, we selected two pepper inbred lines that displayed varying colors for both immature and mature fruits as our research objects. An integrative strategy of transcriptome and metabolome analysis combined with re-sequencing and quantitative determination was adopted to investigate the regulation of color-related genes in pepper fruits.

### 4.1 Decreased chlorophylls and carotenoids should be responsible for light-yellow immature pepper fruit

The formation of various fruit colors in pepper is primarily attributed to the accumulation of chlorophylls, flavonoids and carotenoids in varying proportions (Lightbourn et al., 2008). The pigment determination assay revealed that flavonoids were the predominant pigments in CSJ009Y, whereas the levels of chlorophylls and carotenoids were significantly lower (*P* < 0.01) than those in CSJ010G. This observation prompted us to investigate the differences in the epidermal cells and chloroplasts within the fruit skin between CSJ009Y and CSJ010G.

Chlorophylls and carotenoids are chloroplast-specific pigments, whereas flavonoids are located within the cell vacuoles (Lightbourn et al., 2008). Carotenoid biosynthetic enzymes are predominant in thylakoid membranes (Sun et al., 2018), which are specialized organelles responsible for carotenoid synthesis and sequestration (Nisar et al., 2015). Therefore, maintaining an intact chloroplast structure is crucial for carotenoid and chlorophyll accumulation. The spherical organelles observed in CSJ009Y (marked with green circles in **Figure 3**) highly resembled the progenitors of chloroplasts (proplastids) as described by Mullet (1988). In *Arabidopsis*, the loss of *PIF1* leads to the delayed development of chloroplasts (Pogson and Albrecht, 2011). Similarly, the missense mutation of *PIF1* (*Capana09g001161*) in CSJ009 may impede chloroplast development and subsequently decrease carotenoid and chlorophyll levels in immature pepper fruits. Additionally, the loosely arranged epidermal cells observed in CSJ009Y resembled those observed in the white cucumber fruit skin (Wang et al., 2020). Consequently, a combination of reduced chlorophyll and carotenoids, along with abundant flavonoids contributes to the light-yellow appearance of immature fruits in CSJ009 through the regulation of cell structure and chloroplast development.

Most chlorophyll and carotenoid biosynthesis genes were upregulated in green-fruited CSJ010. Interestingly, most of the identified chlorophyll biosynthesis genes in this study, including *CHLM*, *POR1-like*, *HEMA1*, *CRD1*, and *POR1* (except *CAO*) were downregulated in CSJ009Y compared with CSJ010G. This result further supports the upregulation of most chlorophyll biosynthesis genes in dark green fruits compared to pale green fruits in pepper (Lee et al., 2020). Additionally, *LUT1*, *LCYB*, and *LCYE* have been identified as crucial genes involved in lutein and zeaxanthin formation (Zhang et al., 2020). *CHYB* hydroxylates *β*-carotene to produce zeaxanthin (Diretto et al., 2007). As shown in **Supplementary Table S3**, several carotenoids biosynthesis-related DEGs, including *LUT1*, *CHYB1*, *LCYB2*, and *LCYE1* (except *D27*) were significant upregulated in CSJ010G compared to CSJ009Y. The observed upregulated expression levels of these key genes associated with both chlorophyll and carotenoid biosynthesis may contribute to the higher content of both chlorophyll and carotenoids in CSJ010G relative to CSJ009Y.

Degradation processes also influenced the accumulation of carotenoids. CCD plays a pivotal role in the degradation of various xanthophylls, including zeaxanthin, lutein, and other carotenoids in strawberry (García-Limones et al., 2008), marigold (Zhang et al., 2020) and chrysanthemum (Ohmiya et al., 2006). In contrast, NCED specifically catalyzes the degradation of zeaxanthin (Zhang et al., 2020). In this study, three carotenoid degradation genes *CCD*, *NCED1* and *NCED3*, were upregulated in CSJ010G compared to CSJ009Y. Notably, the upregulation of carotenoid biosynthetic genes observed in green-fruited CSJ010G was accompanied by a simultaneous increase in the expression levels of degradation genes compared to light-yellow-fruited CSJ009Y.

### 4.2 *HCT* may regulate the chlorogenic acid biosynthesis in green pepper fruit

The *HCT* gene is involved in phenylpropanoid biosynthesis (Hoffmann et al., 2004), which regulates chlorogenic acid biosynthesis in tomato (Aseel et al., 2019). In the present study, the total flavonoid content of immature and mature fruits of CSJ010 was significantly higher (*P* < 0.01) than that of CSJ009 (**Figure 1C**). Among the five DEGs identified in the flavonoid biosynthesis pathway in the immature-fruit group (**Supplementary Table S3**), *HCT*, *CCOAOMT6*, *FLS*, and *At4g26220* were all downregulated in CSJ010G vs. CSJ009Y. However, *HCT-like* (*Capana09g000120*) was the only upregulated gene (FPKM = 4.8) in CSJ010G; and no expression (FPKM = 0) was detected in CSJ009Y.

Furthermore, integrative analysis of the metabolome and transcriptome revealed a significant correlation between *HCT-like* gene and chlorogenic acid (MWS0178) levels (**Supplementary Figure S3**). These findings unraveled that *HCT-like* may regulate chlorogenic acid biosynthesis specifically in green-fruited CSJ010 but not yellow-fruited CSJ009 due to the null mutation of *HCT-like* observed in CSJ009. Although chlorogenic acid and chlorophyll content are significantly positively correlated and chlorophyll synthesis interacts with chlorogenic acid synthesis in tobacco leaves (Sheen, 1973), few studies on chlorogenic acid content changing plant fruit color have been reported so far.

### 4.3 *APRR2* may regulate the immature fruit color via reducing carotenoid content

*GLK2*, *LOL1*, *APRR2*, and *PP2C35* (Borovsky et al., 2019; Brand et al., 2014; Lee et al., 2020; Wu et al., 2022) were thought to be related to chlorophyll biosynthesis in immature fruits. WGRS analysis revealed no mutations of *GLK2*, *LOL1* or *PP2C35* in CSJ009. *LOL1* and *GLK* were exclusively detected in the immature-fruit group during the transcriptome analysis (**Table 1**). Furthermore, no significant difference was observed in the expression levels of *GLK2*, *LOL1*, and *PP2C35* between CSJ009 and CSJ010, according to qRT-PCR analysis (**Figure 6**). In contrast, *APRR2* exhibited differential expression within the immature- and mature-fruit groups. Among the four color-related genes analyzed, *APRR2* was the only DEG identified in immature-fruit group (**Table 1**). This finding is consistent with previous results showing that *APRR2* regulates both immature and mature fruit color in pepper (Lee et al., 2020).

*APRR2* has been demonstrated to be closely associated with immature fruit color in a double haploid population (Pan et al., 2013) and an F_2_ population (Lee et al., 2020). *APRR2* regulates the immature fruit color via reducing chloroplast size and thylakoid numbers to decrease the levels of chlorophyll and carotenoids (Lee et al., 2020). However, the role of *APRR2* in governing pepper fruit color appears to be inconsistent. In *C. frutescens*, *APRR2* specifically expressed in immature fruits while being almost undetectable in mature fruits (Jeong et al., 2020). The chlorophyll level of immature fruit in the light-green-fruited line, which harbors a nonsense mutation in *APRR2*, was determined by *CcLOL1* rather than by *APRR2* (Borovsky et al., 2019). Another dark-green-fruited line 165-2 also carried the same nonsense mutation in *APRR2* (Borovsky et al., 2019). These results suggest that reduced chlorophyll content may not be an inescapable consequence of a nonsense mutation in *APRR2*.

WGRS suggests that the nonsense mutation of *APRR2* in CSJ009 is consistent with those found in *C. annuum* ‘IT158782’ (Lee et al., 2020) and *APRR2-Like* gene (Pan et al., 2013), but differs from those observed in *C. frutescens* ‘AC08-201’ (Jeong et al., 2020). The immature fruit color of CSJ009 resembles that of blocky pepper harboring *APRR2-like*, albeit being much paler yellow compared to *C. frutescens* ‘AC08-201’ and *C. annuum* ‘IT158782’. Our quantification demonstrated that the total carotenoid content in the immature fruits of CSJ009 accounted for only 10% of the CSJ010 content. qRT-PCR analysis revealed a significant upregulation of *APRR2* in the immature fruits of CSJ009 compared to CSJ010 (**Figure 6**). Collectively, these findings suggest that *APRR2* may regulate immature fruit color by reducing carotenoid content.

In peppers, the peach fruit has been suggested to be determined by the *c1* locus (Thorup et al., 2000). The regulatory factor *APRR2* regulates mature fruit color via reducing the amount of carotenoids (Lee et al., 2020) and has been recognized as a candidate for the *c1* locus in *C. chinense*, *C. annuum* (Lee et al., 2020), and *C. frutescens* (Jeong et al., 2020). The *c2* (*PSY1*) mutation has been found to be responsible for the ripe orange fruit color in *C. chinense* ‘Habanero Orange’ (Kim et al., 2010). The *c1* (*APRR2*) mutation coupled with the *c2* (*PSY1*) mutation formed peach fruit color in *C. chinense* ‘Habanero Peach’ and no *y* (*CCS*) mutation was found in the line (Lee et al., 2020). The null mutation of *y* (*CCS*), coupled with the nonsense mutation of *c1* (*APRR2*) and the null mutation of *c2* (*PSY1*), produced white mature fruits in *C. annuum* ‘IT158782’ (Lee et al., 2020) and *C. frutescens* ‘AC08-201’ (Jeong et al., 2020). In this study, the mature fruit color of CSJ009 was red, implying the absence of any mutations in *c2* (*PSY1*) and *y* (*CCS*), which was further supported by the WGRS analysis (**Figure 5B**). A premature stop codon was identified in the 8th CDS region of *APRR2* (**Figure 5A**), and the total carotenoid content of red CSJ009 fruit was lower than that of orange CSJ010 fruit (**Supplementary Table S6**), suggesting that the *c1* (*APRR2*) mutation presents in CSJ009. These results indicate that red-fruited CSJ009 contains the *c1C2Y* genotype, orange-fruited CSJ010 harbors the *C1C2y* genotype, peach-fruited pepper has the *c1c2Y* genotype, and white-fruited pepper has the *c1c2y* genotype. In summary, the nonsense mutation of *APRR2* is not an inevitable result of the formation of a mature peach fruit, but a prerequisite.

### 4.4 The null mutation of *CCS* may enhance the biosynthesis of *β,ϵ*-branch carotenoids

The types and levels of carotenoids vary widely among pepper fruits. Red mature CSJ009 fruits predominantly accumulated capsanthin, accompanied by phytoene, *β*-carotene, zeaxanthin palmitate, and violaxanthin myristate (**Supplementary Table S6**). Similarly, mature orange CSJ010 fruits also primarily accumulated lutein, along with phytoene, violaxanthin myristate, lutein palmitate, lutein dimyristate, and *α*-carotene. Phytoene is the main carotenoid in red and orange pepper fruits, next to capsanthin and lutein, thereby challenging previous conclusions that *β*-carotene is the most abundant carotenoid in orange fruits (Guzman et al., 2010), and lutein is usually only found in immature fruits (Gómez-García and Ochoa-Alejo, 2013).

Transcriptional regulation is a major determinant for carotenoid production in the classical model systems of tomato and pepper during fruit ripening in response to developmental signals (Yuan et al., 2015). The accumulation of capsanthin during pepper fruit ripening from green to red is linked with the transcriptional upregulation of *CCS*, along with *PSY*, *PDS*, and *BCH* (Hugueney et al., 1996). Upon comparing the DEGs involved in carotenoid biosynthesis between the mature- and immature-fruit groups, only *CCS* (*Capana06g000615*) and *PSY2* (*Capana02g002284*) were detected in the mature-fruit group. The *PSY2* gene is typically expressed in leaves but also at low levels in ripening fruit in tomato (Paran and van der Knaap, 2007). However, an ortholog of *PSY2* has not yet been identified in pepper, and it has been postulated to exist in pepper and contribute to carotenoid synthesis in mature pepper fruits (Paran and van der Knaap, 2007). In line with this hypothesis, our results demonstrated that the expression of *PSY2* was downregulated in CSJ010O vs. CSJ009R, further supporting its potential role as a key regulator of carotenoid accumulation, specifically in red pepper fruits.

Interestingly, while CSJ009 showed a high expression level of *CCS* (FPKM = 9343), almost no expression was detected in CSJ010 (FPKM = 0.72). The null mutation of *CCS* gene in CSJ010 was identical to the mutations found in CCS-P1 (Jeong et al., 2019) and IT158782 (Lee et al., 2020). The CCS-P1 mutation produces mature orange or yellow fruits (Jeong et al., 2019). Considering the quantification results for capsanthin, it can be inferred that this null mutation in *CCS* leads to the development of orange CSJ010 fruits. Notably, all accessions exhibiting this null mutation belonged to *C. annuum* lines. These results showed that *CCS* was specifically expressed in mature pepper fruits and regulates their red and orange coloration.

*α*-Carotene and *β*-carotene are downstream products of lycopene cyclized by *LCY* genes in *β,ϵ*- and *β,β*-branches, respectively (Zhang et al., 2020). The expression of *LCYB* and *LCYE* has been demonstrated to regulate the bifurcation of the metabolic flux from lycopene to its downstream *β,β*- and *β,ϵ*-branches (Cunningham and Gantt, 1998). The silencing of *LCYE* increased the contents of *β*-carotene in potato tubers (Diretto et al., 2006). While contents of both *β*-carotene and lutein were found to be downregulated when *LCYB* was silenced in tomato (Ma et al., 2011). *LCYB1* (*Capana05g000023*), *LCYB2* (*Capana10g002320*) and *LCYE1* (*Capana09g000177*) have been identified and characterized in pepper, and both *LCYB1* and *LCYB2* had higher expression levels than *LCYE1* in any of the ripening stages (Camara and Dogbo, 1986; Wang et al., 2019). In immature fruits, the *α*-carotene and *β*-carotene contents of CSJ009Y were found to be 12.09% and 6.13% of CSJ010G, respectively. In mature fruits, the *α*-carotene content in CSJ009R (2.61 μg g^-1^ FW) remained lower than that in CSJ010O (18.65 μg g^-1^ FW), while the *β*-carotene content was 4.78 times higher in CSJ009R vs. CSJ010O. The reduced level of *β*-carotene in CSJ009Y is likely attributed to a missense mutation affects *LCYB2* function. Furthermore, the relative expression of *LCYB2* was significantly higher (*P* < 0.01) in CSJ010G than in CSJ009Y (**Figure 6**). These findings strongly suggested that *LCYB2* is impaired in CSJ009 and plays a partial role in carotenoid synthesis in immature pepper fruits. In addition, no *CCS* gene expression was detected in orange CSJ010O; however, a small amount of capsaicin (0.087 μg g^-1^ FW) was still present. This may be attributed to the potential *CCS* activity of *LCYB2* gene, similar to that observed in tomato (Ronen et al., 2000).

The induction of both *LCYB* and *CCS* in competing against *LCYE* involved for synthesizing carotenoids of the *β,β*-branch (Wang et al., 2019). Although the expression of the key carotenoid-biosynthesis genes *CCS* and *LCYB2* was significantly impaired in CSJ010O and CSJ009R, respectively, there was no significant difference in the total carotenoid content between CSJ010O and CSJ009R (**Figure 1F**). Quantification analysis revealed that the major constituents of CSJ009R were carotenoids of the *β,β*-branch, whereas the products of the *β,ϵ*-branch were dominant in CSJ010O. Lutein was the most abundant carotenoid in immature fruits of both CSJ009 and CSJ010, which is consistent with previous studies indicating its prevalence in these stages (Gómez-García and Ochoa-Alejo, 2013). Our quantification demonstrates that the strong expression of *CCS* in CSJ009R resulted in the highest production of capsanthin (104.42 μg g^-1^ FW), while lutein levels significantly decreased to a negligible amount (0.65 μg g^-1^ FW). Conversely, CSJ010O contained lutein as the predominant carotenoid (244.88 μg g^-1^ FW). This is likely attributed to the direct catalytic effect of *CCS* on lycopene and its inhibitory effect on *LCYE* expression in mature pepper fruits (Wang et al., 2019). *CCS* has high sequence identity with *LCYB* and belongs to the lycopene cyclase family (Jeknić et al., 2012). In pepper, the enzyme CCS was found to possess the ability to catalyze lycopene cyclization to produce *β*-carotene (Hugueney et al., 1995). This process was proposed to compete against *LCYE* and repress the synthesis of carotenoids in the *β,ϵ*-branch (Wang et al., 2019). The null mutation of *CCS* leads to a reduced ability for repression of *LCYE* and the biosynthesis of capsanthin in the *β,β*-branch (Lefebvre et al., 1998; Li et al., 2013), thereby promoting the biosynthesis of *α*-carotene and lutein as products of thr *β,ϵ*-branch in CSJ010O.

## Data Availability

The datasets presented in this study can be found in online repositories. Transcriptome data of 12 samples (BioProject accession: PRJNA1095014) have been uploaded to the NCBI (https://www.ncbi.nlm.nih.gov/).
