# Supplementary figures and images for: Multi-omics analysis provides insights into the mechanism underlying fruit color formation in Capsicum
Source: Front Plant Sci. 2024 Nov 6;15:1448060. doi: 10.3389/fpls.2024.1448060 (PMC11576296; doi:10.3389/fpls.2024.1448060)

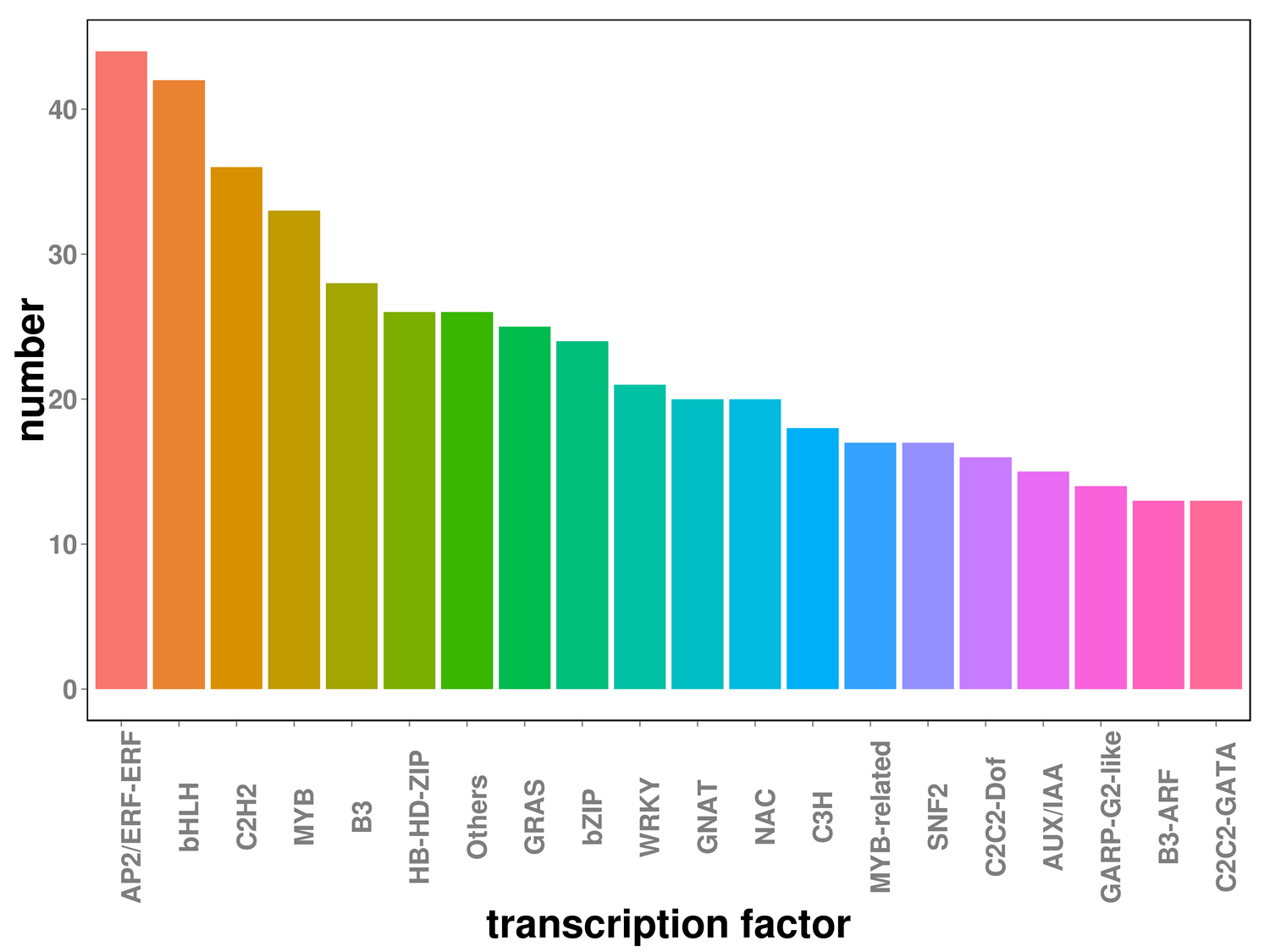

Supplement: Supplementary file 1 [file Image1.tif]

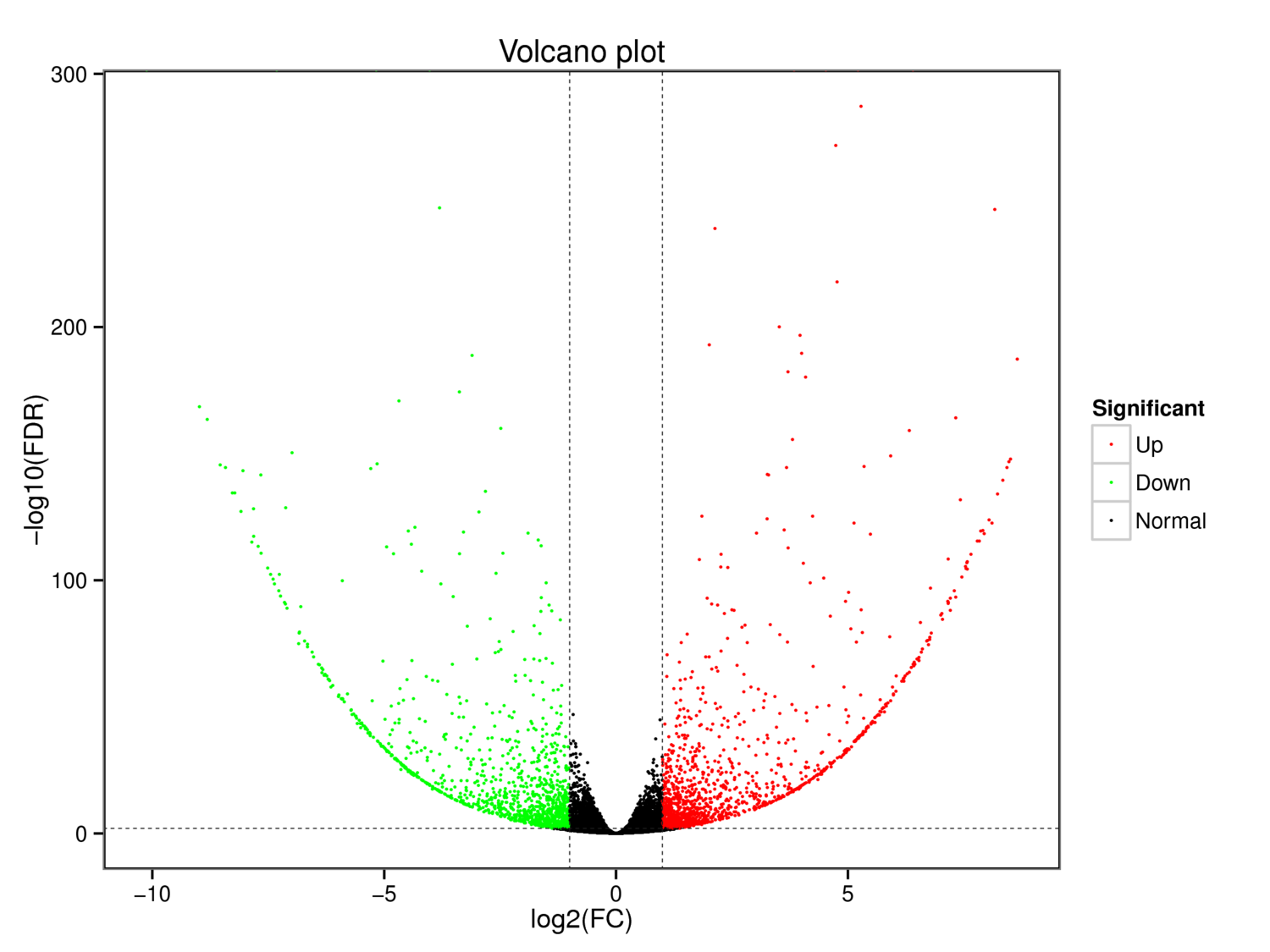

Supplement: Supplementary file 2 [file Image2.tif]

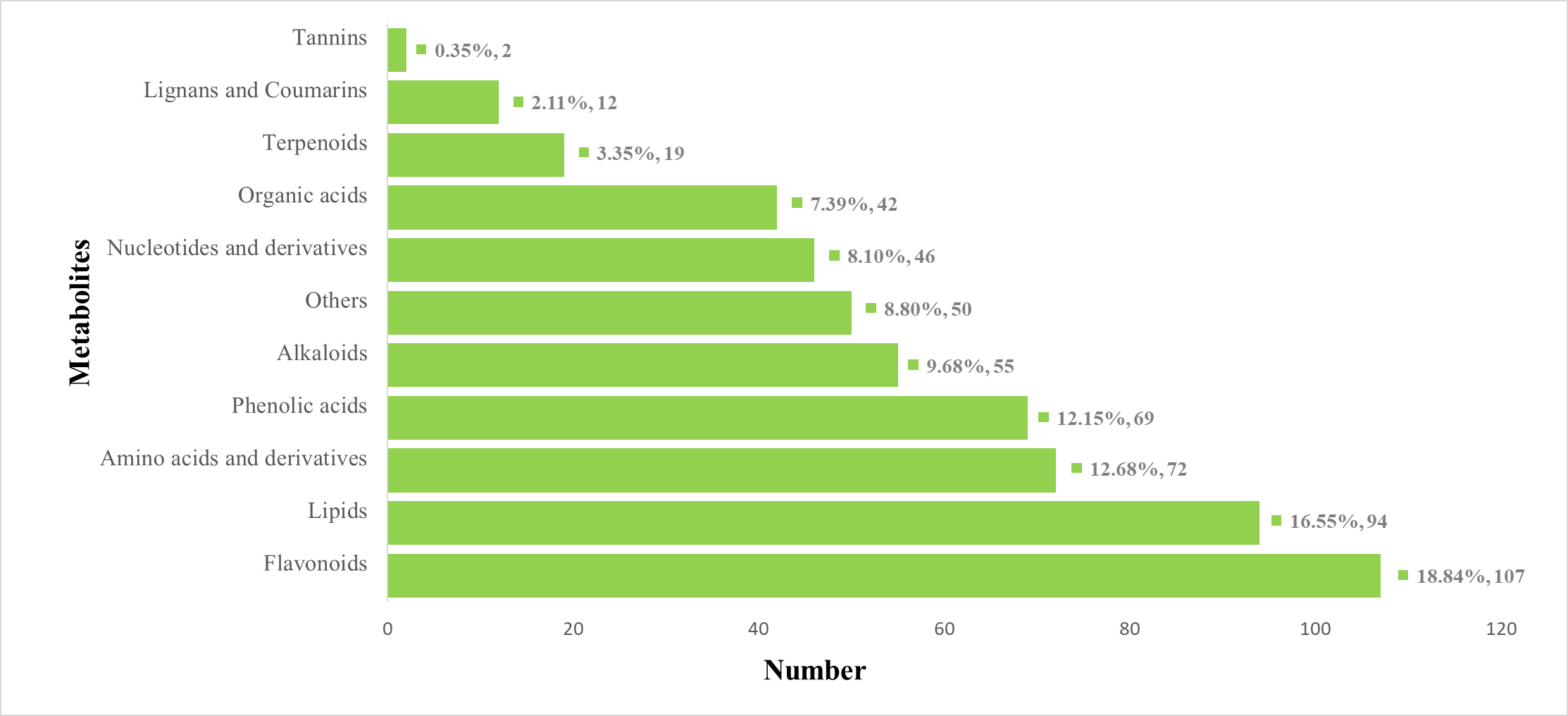

Supplement: Supplementary file 3 [file Image3.tif]
