# Supplementary material for: Multi-omics analysis provides insights into the mechanism underlying fruit color formation in Capsicum
Source: Front Plant Sci. 2024 Nov 6;15:1448060. doi: 10.3389/fpls.2024.1448060 (PMC11576296; doi:10.3389/fpls.2024.1448060)
Supplement: Supplementary file 6 [file Table3.docx]

Table S3 The differential expression genes (DEGs) between CSJ009 and CSJ010 enriched in chlorophyll metabolism, carotenoid metabolism and flavonoid biosynthesis in KEGG analysis.

| Pathway (ID) | Groups | Gene ID | Regulate | Description |
| --- | --- | --- | --- | --- |
| Porphyrin and chlorophyll metabolism (ko00860) | Immature-fruit group (CSJ009Y vs. CSJ010G) | *Capana06g001725* | down | PREDICTED: chlorophyllide a oxygenase, chloroplastic-like [*Capsicum annuum*] |
|  |  | *Capana03g000633* | up | PREDICTED: magnesium protoporphyrin IX methyltransferase, chloroplastic [*Capsicum annuum*] |
|  |  | *Capsicum_annuum_newGene_19011* | down | PREDICTED: chlorophyllase-2, chloroplastic-like [*Solanum tuberosum*] |
|  |  | *Capana10g000065* | up | PREDICTED: protochlorophyllide reductase-like [*Capsicum annuum*] |
|  |  | *Capana04g000804* | up | PREDICTED: glutamyl-tRNA reductase 1, chloroplastic [*Capsicum annuum*] |
|  |  | *Capana04g000513* | up | PREDICTED: uroporphyrinogen-III synthase, chloroplastic [*Capsicum annuum*] |
|  |  | *Capana10g002504* | up | PREDICTED: magnesium-protoporphyrin IX monomethyl ester [oxidative] cyclase, chloroplastic [*Capsicum annuum*] |
|  |  | *Capana00g004560* | up | PREDICTED: protochlorophyllide reductase [*Capsicum annuum*] |
|  | Mature-fruit group (CSJ009R vs. CSJ010O) | *Capana11g000179* | down | lethal leaf spot 1-like protein [*Capsicum annuum*] |
|  |  | *Capana00g004560* | up | PREDICTED: protochlorophyllide reductase [*Capsicum annuum*] |
|  |  | *Capana03g000791* | up | PREDICTED: geranylgeranyl diphosphate reductase, chloroplastic [*Capsicum annuum*] |
|  |  | *Capana11g000180* | down | lethal leaf spot 1-like protein [*Capsicum annuum*] |
|  |  | *Capana01g003169* | up | PREDICTED: divinyl chlorophyllide a 8-vinyl-reductase, chloroplastic [*Capsicum annuum*] |
| Carotenoid metabolism (ko00906) | Immature-fruit group (CSJ009Y vs. CSJ010G) | *Capana01g003704* | up | PREDICTED: 9-cis-epoxycarotenoid dioxygenase NCED3, chloroplastic-like [*Capsicum annuum*] |
|  |  | *Capana11g000423* | up | PREDICTED: abscisic-aldehyde oxidase-like isoform X1 [*Capsicum annuum*] |
|  |  | *Capana10g001912* | up | PREDICTED: carotene epsilon-monooxygenase, chloroplastic [*Capsicum annuum*] |
|  |  | *Capsicum_annuum_newGene_18622* | down | Carotene epsilon-monooxygenase, chloroplastic [*Capsicum chinense*] |
|  |  | *Capana09g000177* | up | PREDICTED: lycopene epsilon cyclase, chloroplastic isoform X1 [*Capsicum annuum*] |
|  |  | *Capana03g002170* | up | beta-carotene hydroxylase 1, chloroplastic [*Capsicum annuum*] |
|  |  | *Capana11g000422* | up | PREDICTED: abscisic-aldehyde oxidase-like isoform X2 [*Capsicum annuum*] |
|  |  | *Capana10g002320* | up | PREDICTED: lycopene beta cyclase, chloroplastic [*Capsicum annuum*] |
|  |  | *Capana**01g000948* | up | PREDICTED: probable carotenoid cleavage dioxygenase 4, chloroplastic [*Capsicum annuum*] |
|  |  | *Capana00g003114* | up | PREDICTED: 9-cis-epoxycarotenoid dioxygenase NCED1, chloroplastic-like [*Capsicum annuum*] |
|  |  | *Capana03g003272* | down | PREDICTED: beta-carotene isomerase D27, chloroplastic isoform X1 [*Capsicum annuum*] |
|  | Mature-fruit group (CSJ009R vs. CSJ010O) | *Capana06g000615* | down | RecName: Full=Capsanthin/capsorubin synthase, chromoplastic; Flags: Precursor |
|  |  | *Capana10g001912* | up | PREDICTED: carotene epsilon-monooxygenase, chloroplastic [*Capsicum annuum*] |
|  |  | *Capana11g000426* | up | PREDICTED: indole-3-acetaldehyde oxidase-like [*Capsicum annuum*] |
|  |  | *Capana11g000428* | up | PREDICTED: abscisic-aldehyde oxidase-like isoform X1 [*Capsicum annuum*] |
|  |  | *Capana02g002284* | down | PREDICTED: phytoene synthase 2, chloroplastic [*Capsicum annuum*] |
| Flavonoid biosynthesis (ko00941) | Immature-fruit group (CSJ009Y vs. CSJ010G) | *Capana03g000549* | down | shikimate O-hydroxycinnamoyltransferase [*Capsicum annuum*] |
|  |  | *Capana00g004448* | down | PREDICTED: probable caffeoyl-CoA O-methyltransferase At4g26220 isoform X1 [*Capsicum annuum*] |
|  |  | *Capana08g002351* | down | caffeoyl-CoA O-methyltransferase 6 [*Capsicum annuum*] |
|  |  | *Capana09g002174* | down | PREDICTED: flavonol synthase/flavanone 3-hydroxylase-like [*Capsicum annuum*] |
|  |  | *Capana09g000120* | up | PREDICTED: shikimate O-hydroxycinnamoyltransferase-like [*Capsicum annuum*] |
|  | Mature-fruit group (CSJ009R vs. CSJ010O) | *Capana02g002586* | down | shikimate O-hydroxycinnamoyltransferase [*Capsicum annuum*] |
|  |  | *Capana09g000119* | up | naringenin,2-oxoglutarate 3-dioxygenase [*Capsicum annuum*] |
|  |  | *Capana00g004448* | down | PREDICTED: shikimate O-hydroxycinnamoyltransferase-like [*Capsicum annuum*] |
|  |  | *Capana10g002482* | up | PREDICTED: probable caffeoyl-CoA O-methyltransferase At4g26220 isoform X1 [*Capsicum annuum*] |
|  |  | *Capana11g000505* | down | PREDICTED: cytochrome P450 98A2-like [*Capsicum annuum*] |

**Gene ID**, the name in the Zunla-1 genome sequence database. **Regulate**, down/up-regulated in the group. **Description**, the annotation in the NR database.
