# Supplementary material for: Multi-omics analysis provides insights into the mechanism underlying fruit color formation in Capsicum
Source: Front Plant Sci. 2024 Nov 6;15:1448060. doi: 10.3389/fpls.2024.1448060 (PMC11576296; doi:10.3389/fpls.2024.1448060)
Supplement: Supplementary file 8 [file Table5.docx]

Table S5 The representative differentially expressed flavonoids (DEFs) identified in immature-fruit group and mature-fruit group

| Group | Class | Compounds | log2FC | Pvalue | VIP | Regulated |
| --- | --- | --- | --- | --- | --- | --- |
| Immature- fruit group (CSJ010G vs. CSJ009Y) | Dihydroflavonol | Hesperetin-5-O-glucoside | -1.01733 | 0.01973 | 1.197 | down |
|  | Dihydroflavone | Eriodictyol-7-O-glucoside | -2.15705 | 0.04313 | 1.218 | down |
|  |  | Hesperetin | -1.96648 | 0.00838 | 1.304 | down |
|  | Flavonoid carbonoside | Luteolin-C-glucosyl-O-sinapic acid | 2.34452 | 0.00237 | 1.324 | up |
|  | Anthocyanins | Cyanidin-3-O-(6''-p-Coumaroylglucoside) | 2.49967 | 0.00398 | 1.283 | up |
|  |  | Delphinidin-3-p-coumaroylrutinoside | -3.21813 | 0.03056 | 1.230 | down |
|  | Flavones | Isorhamnetin-3-O-(6''-malonylglucoside) | 1.05236 | 0.00251 | 1.315 | up |
|  |  | Epicatechin glucoside | -1.08416 | 0.00915 | 1.308 | down |
|  |  | Chrysoeriol-O-sinapoylglucoside | 3.55445 | 0.00002 | 1.335 | up |
|  |  | Tricin-O-saccharic acid | 3.93662 | 0.00431 | 1.325 | up |
|  |  | Apigenin-3-O-rhamnoside | 1.75469 | 0.00777 | 1.291 | up |
|  |  | Luteolin-7-O-neohesperidoside(Lonicerin) | 1.43759 | 0.00740 | 1.243 | up |
|  | Flavonols | Isorhamnetin-3-O-(6''-malonylglucoside)-7-O-rhamnoside | 1.02966 | 0.03020 | 1.162 | up |
|  |  | Isorhamnetin-3-O-(6''-acetylglucoside) | 1.03127 | 0.00653 | 1.319 | up |
|  |  | Kaempferol-3-O-(6''-malonyl)galactoside | -2.57491 | 0.01256 | 1.303 | down |
|  |  | Kaempferol-3-O-galactoside (Trifolin)* | -2.89152 | 0.03141 | 1.253 | down |
|  |  | Syringetin-3-O-glucoside | 1.34455 | 0.00836 | 1.236 | up |
|  |  | Quercetin-3-O-(2''-acetyl)-glucuronide | 1.00444 | 0.04040 | 1.195 | up |
| Mature-fruit group (CSJ009R vs. CSJ010O) | Flavonoid carbonoside | Luteolin-C-glucosyl-O-sinapic acid | 1.27172 | 0.01344 | 1.268 | up |
|  | Anthocyanins | Cyanidin-3-O-(6''-p-Coumaroylglucoside) | 1.42939 | 0.00551 | 1.344 | up |
|  |  | Petunidin-3-p-coumaroylrutinoside | -1.82963 | 0.02121 | 1.315 | down |
|  | Flavones | Kaempferol-3-O-Sambubioside | -1.95638 | 0.04712 | 1.276 | down |
|  |  | Butin-O-glucoside* | -1.80655 | 0.03486 | 1.210 | down |
|  |  | Tricetin | -1.21856 | 0.04194 | 1.268 | down |
|  |  | Chrysoeriol-O-sinapoylglucoside | 3.15848 | 0.02931 | 1.308 | up |
|  |  | Chrysoeriol-O-malonylglucoside | -1.31322 | 0.02406 | 1.335 | down |
|  |  | Tricin-O-saccharic acid | 2.32841 | 0.00014 | 1.391 | up |
|  |  | 3',4',7-Trihydroxyflavone | -2.41228 | 0.04459 | 1.270 | down |
|  |  | Apigenin-7-O-(6'-O-acetyl)-glucoside | -1.06170 | 0.01870 | 1.254 | down |
|  |  | Apigenin-7-(6''-malonylglucoside) | -2.42966 | 0.03538 | 1.186 | down |
|  | Flavonols | Isorhamnetin-3,7-O-diglucoside | 1.26197 | 0.00021 | 1.390 | up |
|  |  | Isorhamnetin-3-O-rutinoside-4'-O-glucoside | 2.53934 | 0.00010 | 1.392 | up |
|  |  | Isorhamnetin-3-O-(6''-malonylglucoside)-7-O-glucoside | 1.75376 | 0.00952 | 1.352 | up |
|  |  | Syringetin-3-O-glucoside | 1.62119 | 0.00126 | 1.370 | up |

DEFs, differentially expressed flavonoids. FC, fold change. log2FC, logarithm of fold change.
