# Supplementary material for: Multi-omics analysis provides insights into the mechanism underlying fruit color formation in Capsicum
Source: Front Plant Sci. 2024 Nov 6;15:1448060. doi: 10.3389/fpls.2024.1448060 (PMC11576296; doi:10.3389/fpls.2024.1448060)
Supplement: Supplementary file 10 [file Table7.docx]

Table S7 The representative differentially expressed carotenoids (DECs) identified from CSJ009 and CSJ010

| Group | Class | Compounds | Pvalue | FC | Log2FC | Type |
| --- | --- | --- | --- | --- | --- | --- |
| CSJ009Y  vs  CSJ009R | carotenes | α-carotene | 0.0065 | 24.3231 | 4.6043 | up |
|  |  | β-carotene | 0.0002 | 31.8257 | 4.9921 | up |
|  | xanthophylls | antheraxanthin dipalmitate | 0.0144 | 17.0960 | 4.0956 | up |
|  |  | lutein dilaurate | 0.0021 | 6.9602 | 2.7991 | up |
|  |  | lutein dimyristate | 0.0010 | 3.7765 | 1.9171 | up |
|  |  | lutein dipalmitate | 0.0138 | 13.7780 | 3.7843 | up |
|  |  | violaxanthin myristate | 0.0005 | 72.9346 | 6.1885 | up |
|  |  | zeaxanthin dimyristate | 0.0003 | 617.829 | 9.2711 | up |
|  |  | zeaxanthin | 0.0013 | 9.6641 | 3.2726 | up |
|  |  | violaxanthin | 0.0006 | 2.7157 | 1.4413 | up |
|  |  | neoxanthin | 0.0003 | 0.3330 | -1.5863 | down |
|  |  | lutein | 0.0007 | 0.0232 | -5.4297 | down |
|  |  | β-cryptoxanthin | 0.0001 | 67.4312 | 6.0753 | up |
|  |  | 8'-apo-beta-carotenal | 0.0000 | 6.0990 | 2.6086 | up |
|  |  | α-cryptoxanthin | 0.0027 | 9.5830 | 3.2605 | up |
| CSJ010G  vs  CSJ010O | carotenes | α-carotene | 0.0010 | 20.9596 | 4.3895 | up |
|  |  | β-carotene | 0.0032 | 0.4084 | -1.2918 | down |
|  | xanthophylls | lutein dilaurate | 0.0002 | 677.411 | 9.4039 | up |
|  |  | lutein dimyristate | 0.0003 | 528.229 | 9.0450 | up |
|  |  | violaxanthin dibutyrate | 0.0005 | 2.3867 | 1.2550 | up |
|  |  | violaxanthin myristate | 0.0010 | 459.954 | 8.8453 | up |
|  |  | antheraxanthin | 0.0134 | 0.1504 | -2.7328 | down |
|  |  | neoxanthin | 0.0002 | 0.0894 | -3.4840 | down |
|  |  | β-cryptoxanthin | 0.0002 | 2.3794 | 1.2506 | up |
|  |  | 8'-apo-beta-carotenal | 0.0004 | 0.4573 | -1.1288 | down |
|  |  | α-cryptoxanthin | 0.0007 | 43.7203 | 5.4502 | up |
|  |  | β-citraurin | 0.0002 | 2.3796 | 1.2507 | up |
| CSJ009Y  vs  CSJ010G | carotenes | α-carotene | 0.0004 | 8.2815 | 3.0499 | up |
|  |  | β-carotene | 0.0018 | 16.3039 | 4.0271 | up |
|  | xanthophylls | lutein dilaurate | 0.0144 | 0.1280 | -2.9661 | down |
|  |  | lutein dimyristate | 0.0053 | 0.1514 | -2.7234 | down |
|  |  | antheraxanthin | 0.0133 | 8.8122 | 3.1395 | up |
|  |  | violaxanthin | 0.0127 | 11.2894 | 3.4969 | up |
|  |  | neoxanthin | 0.0004 | 13.5327 | 3.7584 | up |
|  |  | lutein | 0.0003 | 9.9216 | 3.3106 | up |
|  |  | β-cryptoxanthin | 0.0009 | 7.1752 | 2.8430 | up |
|  |  | 8'-apo-beta-carotenal | 0.0000 | 3.9870 | 1.9953 | up |
| CSJ009R  vs  CSJ010O | carotenes | α-carotene | 0.0004 | 7.1363 | 2.8352 | up |
|  |  | β-carotene | 0.0001 | 0.2092 | -2.2568 | down |
|  | xanthophylls | antheraxanthin dipalmitate | 0.0037 | 6.1038 | 2.6097 | up |
|  |  | lutein dilaurate | 0.0001 | 12.4553 | 3.6387 | up |
|  |  | lutein dimyristate | 0.0003 | 21.1784 | 4.4045 | up |
|  |  | lutein dipalmitate | 0.0000 | 10.5151 | 3.3944 | up |
|  |  | rubixanthin laurate | 0.0005 | 0.1252 | -2.9980 | down |
|  |  | rubixanthin palmitate | 0.0001 | 0.0372 | -4.7502 | down |
|  |  | violaxanthin myristate | 0.0014 | 3.4019 | 1.7663 | up |
|  |  | violaxanthin dilaurate | 0.0023 | 3.8205 | 1.9338 | up |
|  |  | violaxanthin-myristate-laurate | 0.0004 | 4.4975 | 2.1691 | up |
|  |  | zeaxanthin palmitate | 0.0003 | 0.0446 | -4.4882 | down |
|  |  | zeaxanthin-laurate-myristate | 0.0003 | 0.4321 | -1.2107 | down |
|  |  | zeaxanthin dimyristate | 0.0000 | 0.2245 | -2.1553 | down |
|  |  | zeaxanthin-laurate-palmitate | 0.0001 | 0.2113 | -2.2424 | down |
|  |  | zeaxanthin-myristate-palmitate | 0.0008 | 0.0991 | -3.3343 | down |
|  |  | β-cryptoxanthin laurate | 0.0006 | 0.1272 | -2.9749 | down |
|  |  | β-cryptoxanthin myristate | 0.0001 | 0.1025 | -3.2863 | down |
|  |  | zeaxanthin | 0.0015 | 0.3154 | -1.6646 | down |
|  |  | violaxanthin | 0.0015 | 4.8551 | 2.2795 | up |
|  |  | neoxanthin | 0.0053 | 3.6318 | 1.8607 | up |
|  |  | lutein | 0.0005 | 377.417 | 8.5600 | up |
|  |  | β-cryptoxanthin | 0.0001 | 0.2532 | -1.9817 | down |
|  |  | 8'-apo-beta-carotenal | 0.0001 | 0.2989 | -1.7421 | down |
|  |  | capsanthin | 0.0004 | 0.0008 | -10.228 | down |
|  |  | α-cryptoxanthin | 0.0004 | 6.0101 | 2.5874 | up |
|  |  | β-citraurin | 0.0154 | 0.4782 | -1.0643 | down |

DECs, differentially expressed carotenoids. FC, fold change. log2FC, logarithm of fold change.
